# Transcriptome and metabolome analysis of the responses of salt resistance of different *Helianthus annuus* germplasms to melatonin

**DOI:** 10.3389/fpls.2025.1558877

**Published:** 2025-04-16

**Authors:** Changyan Zhao, Yantao Liu, Xiuping Jia, Shengli Liu, Peng Wang, Zhifeng Zhu, Sumei Wan, Wei Duan

**Affiliations:** ^1^ College of Agriculture, Tarim University, Alar, Xinjiang, China; ^2^ Crop Research Institute, Xinjiang Academy of Agricultural and Reclamation Science, Shihezi, Xinjiang, China; ^3^ Crop Research Institute, Gansu Academy of Agricultural Sciences, Lanzhou, Gansu, China; ^4^ China Seed Group Co., Ltd., Sanya, Hainan, China

**Keywords:** growth regulator, *Helianthus annuus*, abiotic stress, multiomics, conjoint analysis

## Abstract

**Introduction:**

Salt stress always causes irreversible damages to the growth of *Helianthus annuus* seedlings in arid and semi-arid areas due to the weakest salt resistance at the seedling stage. Melatonin is a multifunctional molecule that can enhance the salt stress resistance of several crops. However, the effect of melatonin on the salt stress resistance of *H. annuus* is still unclear.

**Methods:**

In this study, four H. annuus germplasms with different salt resistance (YE988, S2102, Longkuiza 4, and 909S) were selected from a total of 164 germplasms from China, France, Chili, the Unit States, etc. Then, four treatments for the four germplasms were designed, including (1) CK, no salt stress + no melatonin application; (2) MT, no salt stress + melatonin application; (3) K, salt stress + no melatonin application; (4) MK, salt stress + melatonin application. After that, the key genes and metabolic pathways involved in the responses of salt resistance of H. annuus germplasms to melatonin were determined by transcriptome and metabolome analysis.

**Results and discussion:**

The results showed that there were 530 differentially expressed genes (37 upregulated genes and 493 down-regulated genes) in *H. annuus* leaves in MK vs. K, and these genes were mainly involved in fatty acids, diterpenoid biosynthesis, linolenic acid metabolism, cysteine and methionine metabolism. There were 60 differentially abundant metabolites (17 up-regulated metabolites and 43 downregulated metabolites) in leaves in MK vs. K, mainly concentrating in tryptophan metabolism, biosynthesis of amino acids, biosynthesis of secondary metabolites and metabolic pathways. The integrated transcriptome and metabolome analysis results showed that melatonin regulated the b-alanine metabolism, monoterpene biosynthesis, and glutathione metabolism pathways, and increased the contents of spermine and spermidine in cells by promoting the expression of genes such as *HannXRQ_Chr07g0195521* and *HannXRQ_Chr03g0093321* in the b-alanine metabolic pathway. In summary, melatonin could enhance salt stress signaling by up-regulating the expression of genes related to the synthesis of spermine and spermidine in *H. annuus* leaves, to regulate photosynthesis and reactive oxygen species metabolism, ultimately enhancing the salt resistance of *H. annuus*. This study will advance understanding of mechanism by which melatonin enhances salt resistance of H. annuus, and provide a scientific basis for the breeding of saltresistant *H. annuus* cultivars.

## 1 Introduction

Soil salinization is a major factor limiting agricultural productivity in arid regions. Saline stress, seriously affecting the growth and development of crops, is mainly caused by accumulation of large amounts of NaCl, Na_2_SO_4_, and other neutral salts. Sodium ions directly enter crop cells through channel and carrier proteins, resulting in ionic toxiciy. In addition, the high sodium ion concentrations outside crop cells reduce the osmotic potential, which drive water molecules out of the cells, resulting in osmotic stress (Li and Yang, 2023). Alkaline stress is caused by accumulation of large amounts of NaHCO_3_ and Na_2_CO_3_. It should be noted that alkaline stress also induces high pH stress. That is, alkaline stress not only causes ionic toxicity and osmotic stress, but also increases pH to interfere with cell pH stability, disrupt cell membrane integrity, and reduce root viability and photosynthetic rate (Zhang et al., 2017; Kaiwen et al., 2020). Many studies have shown that combined saline and alkaline stress leads to severer nutrient imbalance, lower osmoregulatory capacity and antioxidant enzyme activity, and stronger growth inhibition, compared with single saline/alkaline stress (Amirinejad et al., 2017; Wang et al., 2020). In Xinjiang, China, *Helianthus annuus* L. mostly grow in soils with a certain degree of salinization. *Helianthus annuus* has the weakest salt resistance at the seedling stage. Soil salt stress always inhibits *H. annuus* germination and seedling growth, causing great yield losses.

Melatonin is an indole compound that is widely involved in the physiological activities of crops under various abiotic stresses such as salt, drought, extreme temperatures, and heavy metals (Sun et al., 2021). In recent years, the role of melatonin in crops has been intensively studied. For example, melatonin can alleviate the stress-induced growth retardation, senescence, and yield reduction of crops (Ahmad et al., 2023). Melatonin can suppress the accumulation of H_2_O_2_ in maize and *Hevea brasiliensis*, enhancing drought and salt stress resistance (Zhao et al., 2021; Yang et al., 2020). Melatonin can also promote the accumulation of osmolytes such as soluble proteins, proline, peroxidase, and catalase, alleviating osmotic stress in crops (Ren et al., 2022). Previous transcriptome and metabolome analysis results have suggested that melatonin could enhance the cytoprotective and antioxidant mechanisms and salinity resistance of poplar by regulating genes and metabolites involved in photosynthesis, antioxidant enzyme system, and hormone signaling pathways (Li et al., 2024). Liu et al. (2023) found that exogenous melatonin improved the germination rate and germination potential of melon seeds under different conditions by up-regulating the expression of amylase and some melatonin-synthesis genes as well as the activities of antioxidant enzymes and amylase, and reducing the content of malondialdehyde and reactive oxygen species. Duan et al. (2024) found that exogenous melatonin could alleviate the inhibitory effect of alkaline stress on soybean growth and development by regulating the biosynthetic pathways of key compounds terpenes, flavonoids, and phenols. Although melatonin can alleviate the damages of salt stress to some crops, further research is still needed to determine whether melatonin application can alleviate cellular oxidative damage, increase photosynthetic efficiency, and promote the growth of salt-stressed *H. annuus*.

China has a large area of salinized land (36 million hm^2^), of which 9.2 million hm^2^ are arable land (Yuan et al., 2023). As of October 2023, the area of salinized land was about 13.33 million hectares in Xinjiang, accounting for about one-third of the total salinized land in China. The area of salinized arable land is about 1.45 million hectares in Xinjiang, accounting for more than 30% of its total arable land area (https://nynct.xinjiang.gov.cn/). *Helianthus annuus* is an important cash (food or oil-producing) crop in Xinjiang, China (Gehan and Mervat, 2016). Soil salinity is the main factor limiting the vegetative growth of *H. annuus* (Chele et al., 2021). The prominent soil salinization in some areas of Xinjiang seriously restricts the sustainable development of the *H. annuus* industry. According to the 2019 statistics of the Food and Agriculture Organization of the United Nations, China’s *H. annuus* planting area was 850,000 hm^2^, accounting for about 3% of the world’s planting area, ranking sixth in the world (Guo et al., 2021). In 2020, the planting area of *H. annuus* in Xinjiang was 121,400 hm^2^, decreasing by 55.05% compared with that in 2016 (188,200 hm^2^). The output of *H. annuus* seeds in 2020 was 414,000 tons, decreasing by 28.83% compared with that in 2016 (581,700 tons) (China Rural Statistical Yearbook, 2023).

The large area of salinized land in Xinjiang, China poses a great challenge to *H. annuus* production. The use of exogenous substances such as melatonin has become a potential way to enhance the survival, growth, and yield of *H. annuus* under salt stress. Previous studies have used multi-omics to investigate the mechanisms of the salt stress responses of maize (Zhao et al., 2021), poplar (Li et al., 2024), and cantaloupe (Liu et al., 2023) to melatonin treatment, but there is no study on the use of melatonin to improve the salt resistance of *H. annuus* cultivars with different salt resistance. Especially, the physiological and molecular mechanisms of exogenous melatonin regulating the growth of *H. annuus* seedlings under salt stress are still unclear. Therefore, in this study, based on the selected *H. annuus* germplasms with different salt resistance from China, France, Chili, the Unit States, etc., the responses of transcriptome and metabolome of different salt-resistant *H. annuus* cultivars (YE988 < S2102 < Longkuiza 4 < Oil Supreme 909S) to exogenous melatonin under salt stress were analyzed. The objective was to reveal the regulatory mechanism of exogenous melatonin on the salt resistance of *H. annuus* seedlings. This study not only fills the research gap on the role of melatonin in *H. annuus* salt resistance, but also elucidates the mechanism of melatonin regulating *H. annuus* salt resistance through multi omics analysis, providing a sound scientific basis for the breeding of salt-resistant *H. annuus* cultivars and the remediation of salinized soils by agricultural production.

## 2 Materials and methods

### 2.1 Selection of *Helianthus annuus* germplasms with different salt resistance by germination test

*Helianthus annuus* germplasms with different salt resistance including YE988, S2102, Longkuiza 4, and Oil Supreme 909S were selected for germination test (**Table 1**). Seeds were cultured in germination boxes (19 cm × 13 cm × 12 cm), with vermiculite as the substrate. In a pre-test, 0.0796 mol/L (prepared by mixing equal amounts of 0.05 mol/L NaCl solution and 0.0283 mol/L Na_2_CO_3_ solution) was selected as the threshold salt concentration for different *H. annuus* cultivars, by comparing the effects of different salt concentrations on the germination rate, osmolyte accumulation, and antioxidant enzyme activities of *H. annuus* germplasms (**Table 1**, **Supplementary Table S1**). Combined with the measured soil salinity in southern Xinjiang (NaCl: NaCO_3_ = 1: 0.97), salt solution was prepared by mixing 3 g of NaCl, 3 g of NaCO_3_, and distilled water for the present test.

**Table 1:** Effects of different salt concentrations on the germination rate of *Helianthus annuus* seeds.

Cultivar	Source	Salt concentration (mol·L^-1^)	Germination rate (%)
Oil Supreme 909S	Beijing Duolefu Agricultural Technology Center, China	0	100
		0.0398	100
		0.0796	85
		0.1194	70
Longkuiza 4	Heilongjiang Academy of Agricultural Sciences, China	0	96
		0.0398	92
		0.0796	76
		0.1194	44
S2102	China Seed Company (France)	0	92
		0.0398	84
		0.0796	40
		0.1194	20
YE988	Chifeng Nianfeng Seed Industry Co., Ltd, China	0	92
		0.0398	76
		0.0796	16
		0.1194	0

Oil Supreme 909S has the strongest salt resistance, followed by Longkuiza 4, S2102, and YE988.

### 2.2 Experimental design

There were a total of four treatments (**Table 2**), and each treatment had six replicates. The experiment was carried out in a climatic chamber of the Key Laboratory of Cereal Genetics of Xinjiang Production and Construction Corps in Xinjiang Academy of Agricultural and Reclamation Science, Shihezi, China (temperature: 24 – 30°C; humidity: 45% – 50%; light intensity: 1 – 20,000 lx). Seeds with consistent size, undamaged seed coat, and intact embryos were sterilized with 75% ethanol, washed with distilled water, and evenly placed in a culture tray with moist filter paper in an incubator. After one week, the stems below the cotyledons of *H. annuus* seedlings were wrapped with a sponge, and moved in plastic plates with holes. The plastic plates were placed in basins containing nutrient solution (945 mg/L of calcium nitrate, 607 mg/L of potassium nitrate, 115 mg/L of ammonium phosphate, 493 mg/L of magnesium sulfate, 2.5 mL/L of ferric salt solution, and 5 mL/L of trace elements). After the seedlings had two true leaves, twelve seedlings were transplanted to a germination box (20 cm × 14 cm × 8 cm) with 225 g of vermiculite and 500 mL of distilled water/salt solution, and placed in a climate chamber. A randomized block experimental design was used to assign a total of 48 germination boxes to four treatments. In a pre-test, the effects of different concentrations of melatonin on the germination rate of *H. annuus* seeds under salt stress was explored. It was found that foliar spraying with melatonin at the concentration of 10 μmol·L^-^¹ for three consecutive days resulted in significant differences compared with the control (**Supplementary Table S2**). Therefore, in this experiment, melatonin provided by Beijing Solaibao Technology Co., Ltd., China with a purity of 99.0% was used, i.e., 0.05 g of melatonin was dissolved in 1 mL of anhydrous ethanol, and then distilled water was added to make the volume to 1 L, to prepare the melatonin solution with a melatonin concentration of 10 μmol·L^-1^.Starting from the day of salt solution addition, melatonin was sprayed on *H. annuus* seedling leaves using a 100 mL sprayer at 21: 00 for three consecutive days. Melatonin solution was evenly sprayed on both sides of the seedling leaves, until a water film was formed on the leaves and no dripping occurred. Plant leaves were sampled on the 4th day (n = 3), and stored in liquid nitrogen for transcriptome and metabolome analysis (the number of biological replicates for each treatment was three in transcriptomic analysis and six in metabolomic analysis).

**Table 2:** Experimental design.

Treatment	Salt solution (mol·L^-1^)	Melatonin solution (μmol·L^-1^)
CK	0	0
MT	0	10
K	0.0796	0
MK	0.0796	10

CK, no salt stress + no melatonin application; MT, no salt stress + melatonin application; K, salt stress + no melatonin application; MK, salt stress + melatonin application.

### 2.3 Measurements

#### 2.3.1 Total RNA isolation and transcriptome analysis

RNA was extracted from plant tissues using the RNA Extraction Kit (Thermo Fisher Scientific Co., Ltd., China) according to the instructions of the manufacturer, followed by quality control of RNA samples using the Agilent 2100 bioanalyzer (Agilent, China) to precisely detect the RNA integrity. Ribosomal RNA was removed from the total RNA, to obtain mRNA. Subsequently, the mRNA was randomly broken with divalent cations in the NEB fragmentation buffer. A library was prepared according to the NEB normal or strand-specific library preparation method (Parkhomchuk et al., 2009). The prepared library was quantified using the Qubit2.0 Fluorometer (Thermo Fisher Scientific, USA), diluted to 1.5 ng/ul, followed by the detection of the insert size using the Agilent 2100 bioanalyzer. After library inspection, sequencing was performed on different libraries on the Illumina platform. Reference genome and gene annotation files were downloaded directly from the genome website (ensemblplants_helianthus_annuus_hanxrqr1_0_gca_002127325_1). HISAT2 software (v2.0.5) was used to construct an index of the reference genome, and paired clean reads were aligned with the reference genome using HISAT2 (v2.0.5) for featureCounts. DESeq2 software (1.20.0) was used for differential analysis. DESeq2 provides a statistical procedure for determining differential expression in digital gene expression data using a model based on a negative binomial distribution. The Benjamini-Hochberg method (Ferreira et al., 2008) was used to adjust the *p* value to control the false discovery rate. Genes with fold change ≥ 1 and *p* value ≤ 0.05 were considered differentially expressed genes (DEGs). Cluster Profiler software was used to perform Gene Ontology (GO) functional and Kyoto Encyclopedia of Genes and Genomes (KEGG) pathway enrichment analysis on the DEGs, with *p* < 0.05 as the cutoff for determining significant enrichment.

Normalization and differential analysis of the RNA-seq data were completed in DESeq2 software (1.20.0). The gene expression data was normalized using a negative binomial distribution model in DESeq2. The total number of sequencing reads of the sample was considered during data processing, the size factor for each sample relative to a reference sample was calculated, and the original count of each gene was divided by the size factor of the sample, to achieve normalization of the data. This method can effectively eliminate differences due to different sequencing depths and accurately identify DEGs. Pairwise comparison of transcription levels in *H. annuus* leaves under different conditions was performed to explore the gene expression differences in MT *vs.* CKs, K *vs.* MK, as well as between different salt-resistant *H. annuus* cultivars within each treatment. This aimed to reveal the mechanism by which exogenous melatonin improves the salt resistance of *H. annuus.* RMATS software was used for the quantification and differential analysis of alternative splicing events.

#### 2.3.2 Metabolome analysis

Non-targeted metabolomic analysis were carried out based on liquid chromatography–mass spectrometry (LC–MS) (Dunn et al., 2011; Benson et al., 2023). The experimental procedure mainly included metabolite extraction, LC–MS/MS detection, and data analysis. The first three QCs were used to correct chromatography-mass spectrometry system before sample injection. The next three QCs were scanned in segments and used for the characterization of metabolites, together with the secondary spectral acquisition from the samples. The QCs used in the middle stage of the sample detection were used to evaluate the stability of the system and perform quality control analysis on the data. Firstly, the raw data (.raw) obtained by mass spectrometry detection was imported into Compound Discoverer 3.3 software for spectral processing and database search, to obtain the qualitative and quantitative results of metabolites. For data normalization and quality control, peak extraction was conducted after setting quality deviation (5 ppm), signal strength deviation (30%), minimum signal strength, etc., followed by the annotation with mzCloud (https://www.mzcloud.org/), mzVault, and Masslist databases. Reliable metabolites were selected based on the annotation of mass spectrometry data (mass-to-charge ratio, retention time, fragment ions) with the above public databases. Based on the comprehensive information of multiple databases, the metabolites were divided into 11 major categories (such as amino acids, lipids, and terpenes). Then, the raw quantitative results were normalized according to the formula: Raw quantitative value of the sample metabolites/(total quantitative value of sample metabolites/total quantitative value of QC1 sample metabolites). After that, metabolites with a coefficient of variation (CV) less than 30% in QC samples (experimental samples mixed by equal volume) were retained as the final identification results for subsequent analysis (Zhang et al., 2024). Next, multivariate statistical analyses of metabolites, including principal component analysis (PCA) and partial least squares discriminant analysis (PLS-DA), were performed to reveal the differences in metabolic patterns between different treatments. The differentially abundant metabolites (DAMs) were selected using three parameters including VIP (variable importance in the projection), FC (fold change), and *p* value (t-test), i.e., VIP > 1.0; FC > 1.2 or FC < 0.833; *p* value < 0.05 (Haspel et al., 2014; Heischmann et al., 2016). The KEGG analysis was performed on DAMs (Jia et al., 2016; Picart-Armada et al., 2018). Hierarchical clustering and correlation analysis were conducted to reveal relationships between samples and between metabolites. The biological significance of metabolites was clarified through functional analysis of metabolic pathways. Identified metabolites were subjected to functional annotation and classification using databases include Kyoto Encyclopedia of Genes and Genomes (KEGG), Human Metabolome Database (HMDB), and LIPID MAPS Database, to understand the functional characteristics and classification of different metabolites.

False positives were minimized through strict FDR control (adjusted *p* ≤ 0.05) and integration of multi-omic data. The reliability of DEGs was ensured through bio-function verification (such as KEGG pathway enrichment analysis) and metabolite association analysis. This strategy captures subtle expression changes while ensuring statistical rigor. To better understand the interaction between the transcriptome and metabolome of salt-stressed *H. annuus* under melatonin application, DEGs and DAMs from different comparison groups were simultaneously mapped to the KEGG database. Then, the relationships between DAMs and DEGs were intuitively presented through the correlation network, and the top 10 DEGs by expression level and the top 5 DAMs by relative abundance were selected for plotting, followed by the plotting of KEGG pathways to obtain the pathways simultaneously enriched by the DAMs and DEGs. After that, key DEGs and DAMs were identified from key pathways such as β-alanine metabolism, monoterpene biosynthesis, and glutathione metabolism.

### 2.4 Statistic analysis

DESeq2 (1.20.0) and clusterProfiler (3.8.1) were used for differential analysis of transcriptome and gene set enrichment analysis. R (Version 3.5.0) was used for graphing. The identified metabolites were annotated using the KEGG database (https://www.genome.jp/kegg/pathway.html), the Human Metabolome Database (HMDB) database (https://hmdb.ca/metabolites), and the Lipid Metabolites and Pathways Strategy (LIPIDMaps) database (http://www.lipidmaps.org/). PCA and PLS-DA were performed after transforming raw data using metaX in R software. Volcanic maps were plotted using the ggplot2 package in R software, heatmaps were plotted using the Pheatmap package, and correlation networks were plotted using the corrplot package.

Values are represented as mean ± SD (standard deviation). Where appropriate, the statistical significance of differences was determined by the t-test or one-way ANOVA with Bonferroni’s *post hoc* comparisons, and the differences with *p*-value less than 0.05 were considered significant. SPSS version 26.0 (IBM Corp., Armonk, NY, USA) was used for statistical analysis.

## 3 Results

### 3.1 Differentially expressed genes in salt-stressed *H. annuus* leaves under exogenous melatonin application

**Figure 1:**
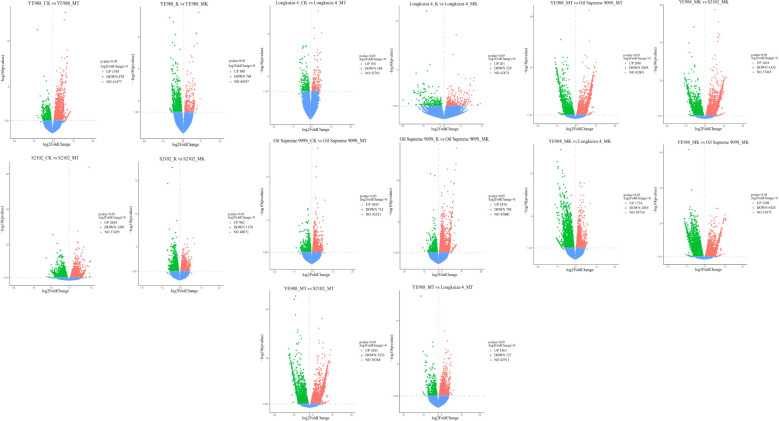
Differentially expressed genes in different comparison groups (*p* < 0.05). Blue dots represent the genes with no significant differences in expression between treatments, and orange and green dots indicate genes that are significantly up-regulated and down-regulated, respectively. CK, no salt stress + no melatonin application; MT, no salt stress + melatonin application; K, salt stress + no melatonin application; MK, salt stress + melatonin application. The same below.

For cultivar YE988, there were 1873 DEGs in MT *vs.* CK (1395 up-regulated genes and 478 down-regulated genes), and 1077 DEGs in K *vs.* MK (308 up-regulated genes and 769 down-regulated genes) (**Figure 1**). For cultivar S2102, there were 6135 DEGs in MT *vs.* CK (2644 up-regulated genes and 3491 down-regulated genes), and 2321 DEGs in K *vs.* MK (962 up-regulated genes and 1359 down-regulated genes). For cultivar Longkuiza 4, there were 381 DEGs in MT *vs.* CK (193 up-regulated genes and 188 down-regulated genes), and 490 DEGs in K *vs.* MK (261 up-regulated genes and 229 down-regulated genes). For cultivar Oil Supreme 909S, there were 1745 DEGs in MT *vs.* CK (1033 up-regulated genes and 712 down-regulated genes), and 2581 DEGs in K *vs.* MK (1876 up-regulated genes and 705 down-regulated genes).

In the MT treatment, there were 7748 DEGs in S2102 *vs.* YE988 (3418 up-regulated genes and 4330 down-regulated genes), 2619 DEGs in Longkuiza 4 *vs.* YE988 (1734 up-regulated genes and 2885 down-regulated genes), and 11733 DEGs in Oil Supreme 909S *vs.* YE988 (5309 up-regulated genes and 6424 down-regulated genes). In the MK treatment, there were 7724 DEGs in S2102 *vs.* YE988 (4201 up-regulated genes and 3523 down-regulated genes), 2190 DEGs in Longkuiza 4 *vs.* YE988 (1463 up-regulated genes and 727 down-regulated genes), and 4696 DEGs in Oil Supreme 909S *vs.* YE988 (2601 up-regulated genes and 2095 down-regulated genes) (**Figure 1**).

### 3.2 Enrichment analysis of differentially expressed genes in salt-stressed *H. annuus* leaves under melatonin treatment

**Figure 2:**
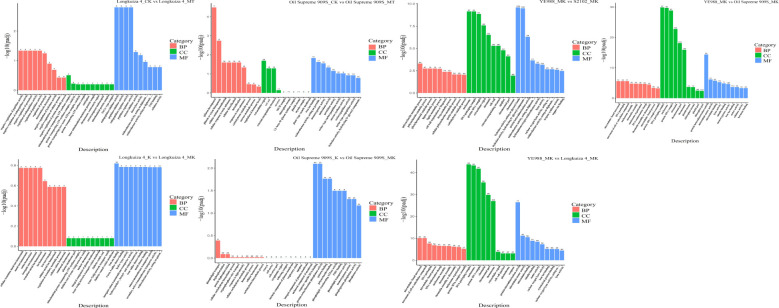
Gene ontology (GO) analysis of differentially expressed genes (DEGs). The abscissa represents GO terms, and the ordinate represents the significance level of GO term enrichment, with higher values indicating greater significance. The values on the column represented the number of enriched DEGs.

The Gene Ontology (GO) analysis of DEGs showed that the functions of DEGs could be divided into three categories and thirty subcategories (**Figure 2**; **Supplementary Figure S1**). The DEGs (604 genes in total) in cultivar YE988 in K *vs.* MK were mainly enriched in the Cellular component category, accounting for 40.78% of the total number of annotated genes. The DEGs (876 genes in total) in cultivar S2102 in K *vs.* MK were mainly enriched in sequence-specific DNA binding, oxidoreductase activity, carbohydrate binding, heme binding, monooxygenase activity, and iron ion binding, accounting for 32.9% of the total number of annotated genes. The DEGs (164 genes in total) in cultivar Longkuiza 4 in K *vs.* MK were mainly enriched in Biological process and Molecular function categories, accounting for 84% of the total number of annotated genes. The DEGs (394 genes in total) in cultivar Oil Supreme 909S in K *vs.* MK were mainly enriched in the Molecular function category, accounting for 44% of the total number of annotated genes.

**Figure 3:**
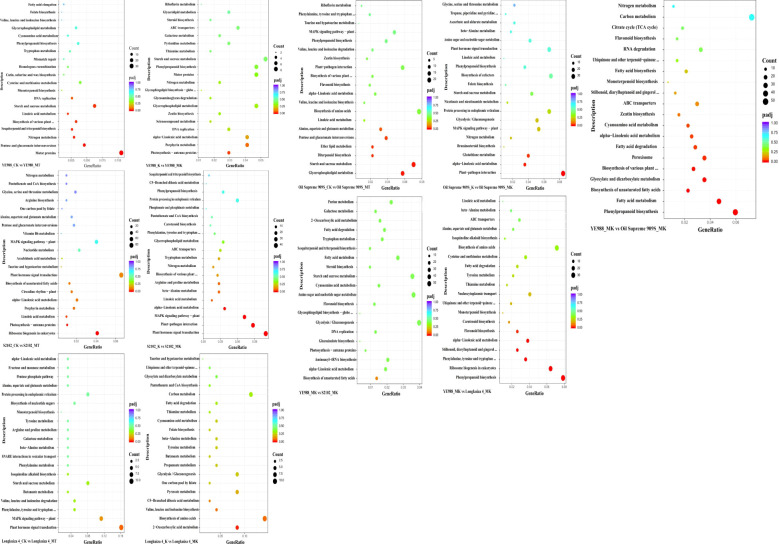
Kyoto Encyclopedia of Genes and Genomes (KEGG) enrichment analysis of differentially expressed genes (DEGs). The abscissa represents the ratio of the number of DEGs annotated on GO (Gene ontology) terms to the total number of DEGs, and the ordinate represents GO terms. The size of the dots represents the number of DEGs annotated on GO terms, and the colors from red to purple represent the significance of enrichment from high to low.

In the MT treatment, S2102 *vs.* YE988 had the most DEGs, and the number of DEGs of Oil Supreme 909S *vs.* YE988 enriched in Cellular component was the least. In the MK treatment, melatonin affected the functional genes of the Cellular component category in different salt-resistant *H. annuus* cultivars. The suppression of melatonin on the expression of Cellular component genes increased with the increase of *H. annuus* salt resistance. However, in the MK treatment, the expression of Cellular component genes increased with the increase of *H. annuus* salt resistance after melatonin application. There were more changes in Cellular component genes than in other genes in different comparison groups. The KEGG enrichment analysis found that the DEGs were mainly enriched in Fatty acid biosynthesis, Alpha-Linolenic acid metabolism, Pentose and glucuronate interconversions, Diterpenoid biosynthesis, Cysteine and methionine metabolism, and Motor proteins pathways (**Figure 3**; **Supplementary Figure S2**). Therefore, melatonin up-regulated the genes related to fatty acid and diterpenoid biosynthesis, linolenic acid metabolism, and cysteine and methionine metabolism, to improve the salt resistance of salt-stressed *H. annuus* seedlings.

Effect of alternative splicing on the function of *GAUT6*: *GAUT6* is involved in the pectin synthesis in cell wall, affecting cell structural stability. It indicates that melatonin may enhance cell wall integrity by regulating the splicing pattern of GAUT6, enhancing salt resistance. For the splicing event for unknown functional genes, in the *HannXRQ_Chr00c0486g0575011* treatment, only the inclusion-type expression (68,91,65) was detected, and the jump-ype was 0, indicating that the splicing event may be specifically activated by melatonin and may involve unknown salt-resistant related functions (**Table 3**).

**Table 3:** Results of analysis of variance of alternative splicing events.

GeneID	geneSymbol	chr	strand	exonStart_0base	exonEnd	IJC_SAMPLE_1	SJC_SAMPLE_1	IncFormLen	SkipFormLen
HannXRQ_Chr00c0494g0575111	GAUT6	HanXRQChr00c0494	-	14478	14620	19,30,3	15,8,0	291	149
HannXRQ_Chr00c0486g0575011	NA	HanXRQChr00c0486	-	5540	5710	68,91,65	0,0,0	319	149
HannXRQ_Chr00c0117g0571731	NA	HanXRQChr00c0117	-	23985	24305	91,80,141	0,0,0	469	149
HannXRQ_Chr00c0117g0571731	NA	HanXRQChr00c0117	-	25735	25835	69,26,76	2,1,0	249	149
HannXRQ_Chr00c0070g0571571	NA	HanXRQChr00c0070	+	38921	38948	0,0,0	0,1,0	176	149

GeneID, Number of the gene where the alternative splicing event is located; geneSymbol, Name of the gene where the alternative splicing event is located; chr, Chromosome where the alternative splicing event is located; strand, Direction of the chromosome chain where the alternative splicing event is located; exonStart_0base, The start position of the exon skip of the alternative splicing event, counting from 0; exonEnd, The end position of the exon skip of the alternative splicing event; IC_SAMPLE_1: The expression of the alternative splicing event Exon Inclusion Isoform in the treatments in pair-wise comparisons; SC_SAMPLE_1: The expression of the alternative splicing event Exon Skipping Isoform in the treatments in pair-wise comparisons; IncFormLen: Effective length of the alternative splicing event Exon Inclusion Isoform; SkipFormLen, Effective length of the alternative splicing event Exon Skipping Isoform.

### 3.3 Differentially abundant metabolites in salt-stressed *H. annuus* leaves under melatonin treatment and KEGG analysis

**Figure 4:**
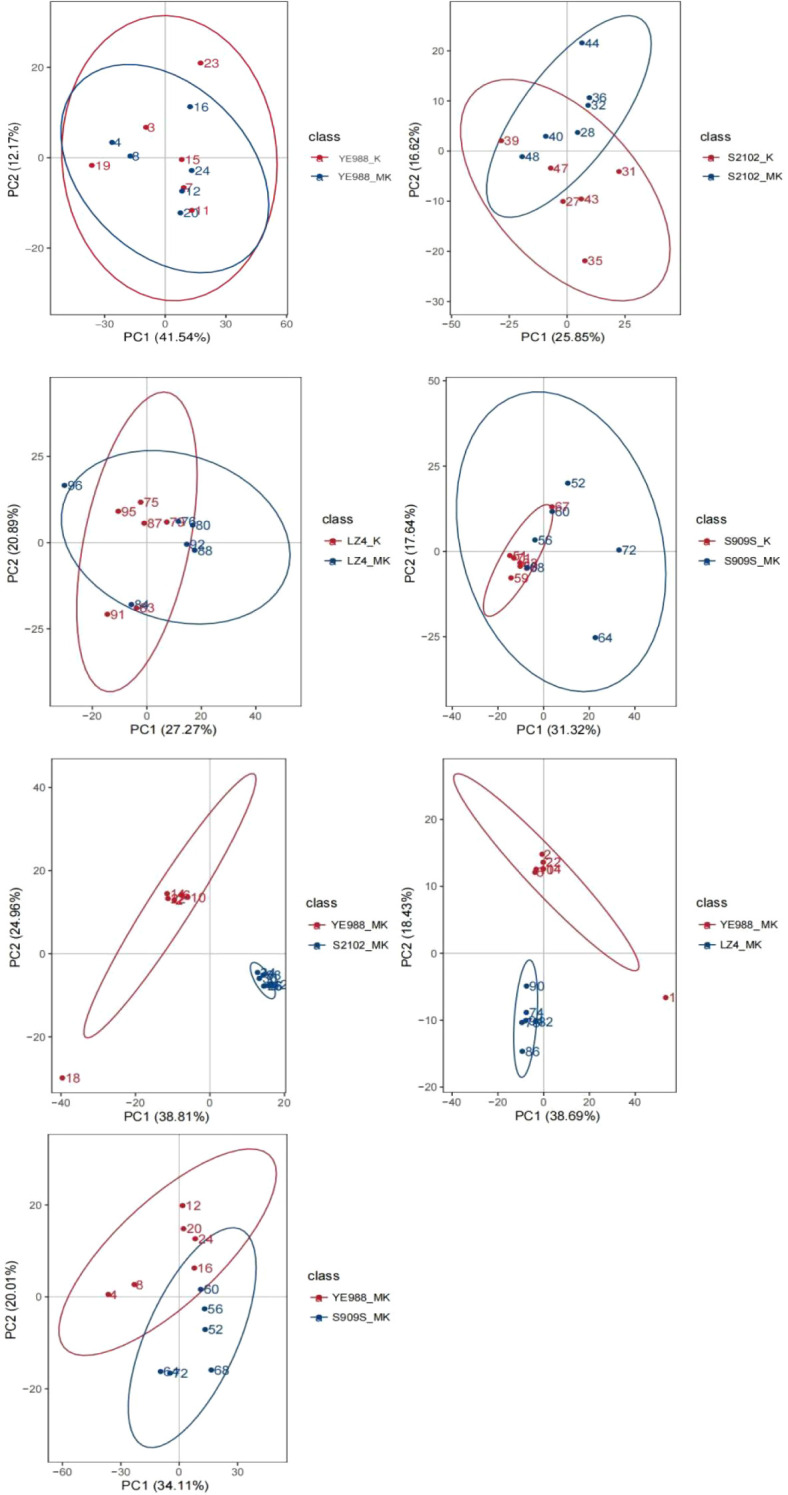
Principal component analysis (PCA) of differentially abundant metabolites. The abscissa PC1 and ordinate PC2 in the figure represent the scores of the first and second principal components, respectively. The points of different colors represent the samples of different comparison groups. The ellipses represent 95% confidence interval.

The PCA (**Figure 4**) illustrated the overall changes in DAMs between different treatments. The first principal component (PC1) explained the largest variance in the data, while the second principal component (PC2) explained the second largest variance. The separation of points along PC1 and PC2 indicated different metabolic profiles among the treatments. The clear separation between K and MK treatments indicated that melatonin significantly altered the metabolite composition in *H. annuus* leaves. The overlapping parts between different treatments indicate the same DAMs, with smaller overlaps indicating more DAMs. The PCA found that cultivars YE988, S2102, Longkuiza 4, and Oil Supreme 909S had small intra-group differences and large inter-group differences, indicating the reliability of the data.

**Figure 5:**
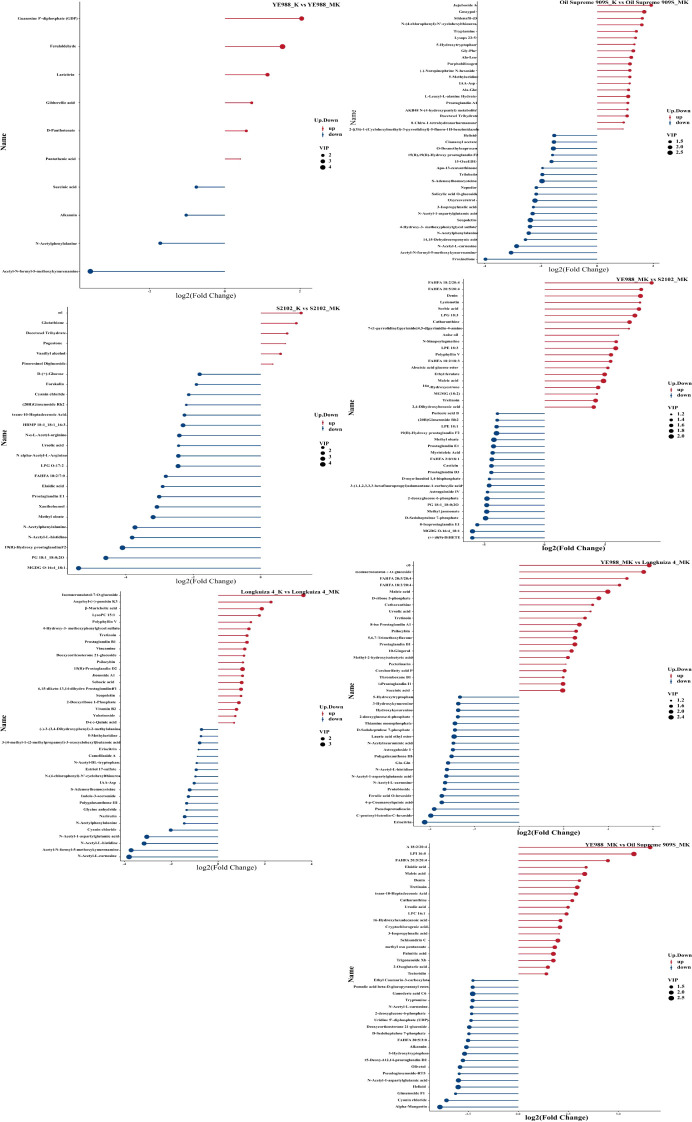
Matchstick diagram of the top twenty differentially abundant metabolites by abundance. Blue dots represent down-regulation, and red dots represent up-regulation; The length of the sticks represents the magnitude of the *p*-value; The size of the dot represents the magnitude of the VIP (variable importance in the projection) value.

The identified 1184 DAMs were classified into eleven categories. In CK *vs.* MT, Longkuiza 4 experienced the most significant changes among the four cultivars, i.e., there were 28 up-regulated and 78 down-regulated DAMs in cultivar Longkuiza 4. In K *vs.* MK, the changes of DAMs in cultivars S2102, S909S, and longkuiza 4 were significantly larger than those in CK *vs.* MT. Cultivar S2102 had 12 up-regulated DAMs and 69 down-regulated DAMs. Cultivar S909S had 42 up-regulated metabolites and 96 down-regulated metabolites. Cultivar Longkuiza 4 had 29 up-regulated metabolites and 33 down-regulated metabolites. There were significant changes in metabolites in YE988, S2102, Longkuiza 4, and Oil Supreme 909S after foliar spraying with melatonin, with the greatest difference observed in S2102 *vs.* YE988. In the MT treatment, there were 185 up-regulated metabolites and 160 down-regulated metabolites in YE988 *vs.* S2102. In the MK treatment, there were 190 up-regulated metabolites and 200 down-regulated metabolites in YE988 *vs.* S2102 (**Table 4**). In K *vs.* MK, the up-regulated DAMs of cultivar YE988 were Guanosine 5’-diphosphate (GDP) and Ferulaldehyde, and the down-regulated DAM was Acetyl-N-formyl-5-methoxykynurenamine. The up-regulated metabolites of cultivar S2102 were Eugenol and Glutathione, and the down-regulated metabolites were PG 18:1_18:0;20 and MGDG O-16:4_18:1. The up-regulated metabolites of cultivar Longkuiza 4 were Jujuboside, Gossypol, Sildenafil-d3, and N-(4-chlorophenyl)-N’-cyclohexylthiourea, and the down-regulated metabolites were N-Acetyl-L-carnosine, Acetyl-N-formyl-5-methoxykynurenamine, and Fraxinellone. The up-regulated metabolites of cultivar Oil Supreme 909S were Isomucronulatol-7-O-glucoside, Angeloyl-(+)-gomisin K3, and β-Muricholic acid, and the down-regulated metabolites were Acetyl-N-formyl-5-methoxykynurenamine and N-Acetyl-L-carnosin (**Figure 5**).

**Table 4:** Statistics of differentially abundant metabolites.

Comparison group	Total number of metabolites	Up-regulated metabolites	Down-regulated metabolites
YE988-CK *vs.* YE988-MT	1184	10	45
S2102-CK *vs.* S2102-MT		2	23
Oil Supreme 909S-CK *vs.* Oil Supreme 909S-MT		0	21
Longkuiza 4-CK *vs.* Longkuiza 4-MT		28	78
YE988-K *vs.* YE988-MK		4	25
S2102-K *vs.* S2102-MK		12	69
Oil Supreme 909S-K *vs.* Oil Supreme 909S-MK		42	96
Longkuiza 4-K *vs.* Longkuiza 4-MK		29	33
YE988-MK *vs.* S2102-MK		190	200
YE988-MK *vs.* Oil Supreme 909S-MK		108	96
YE988-MK *vs.* Longkuiza 4-MK		137	123
YE988-MT *vs.* S2102-MT		185	160
YE988-MT *vs.* Oil Supreme 909S-MT		86	53
YE988-MT *vs.* Longkuiza 4-MT		98	138

**Figure 6:**
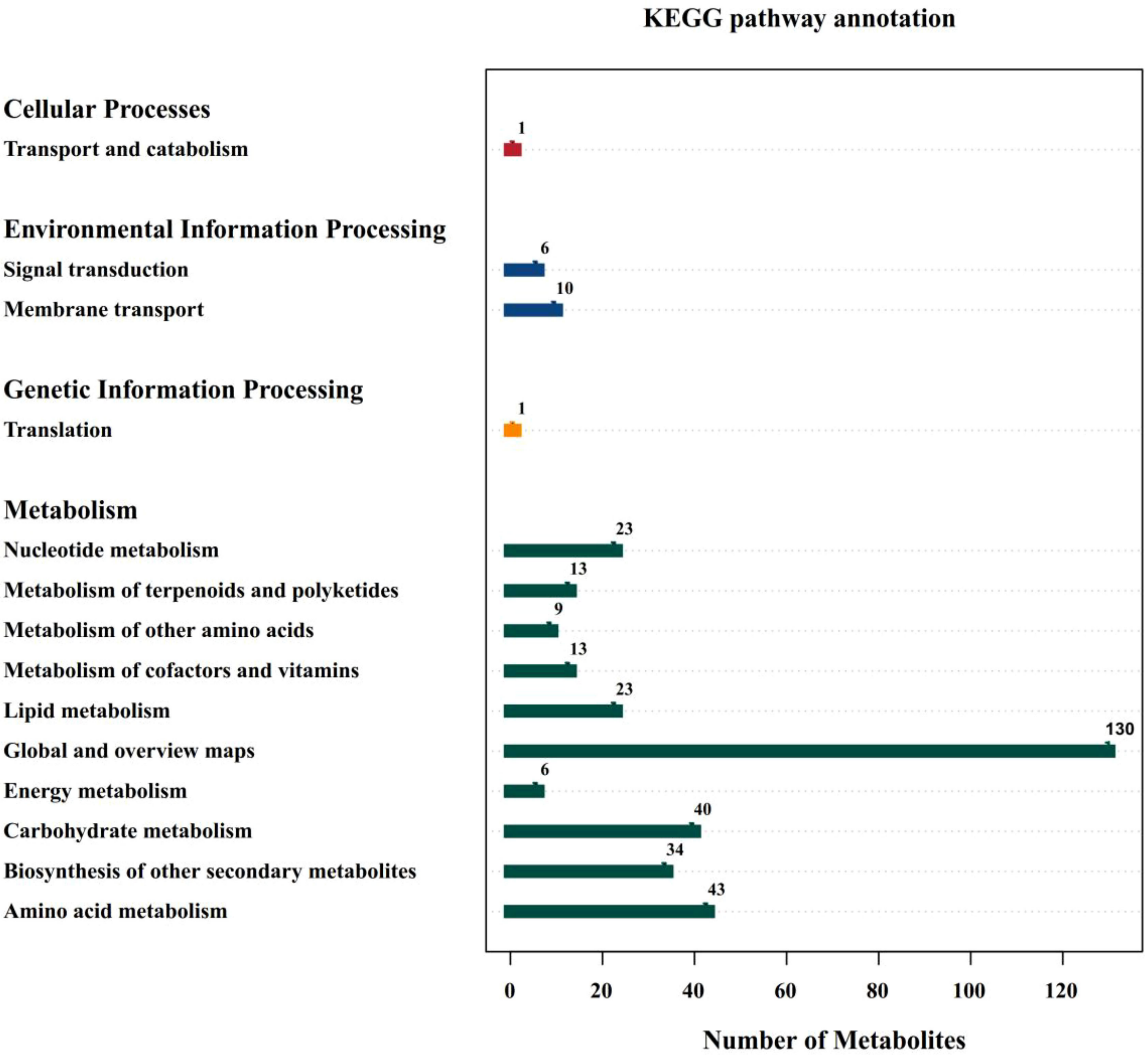
Kyoto Encyclopedia of Genes and Genomes (KEGG) pathway annotation. The abscissa represents the number of differentially abundant metabolites, and the ordinate represents the annotated KEGG pathways.

Under salt stress (MK treatment), the up-regulated metabolites were TG 16:0_18:2_18:2 and Oleoyl ethylamide in YE988 *vs.* S2102, and the down-regulated metabolites were Carbendazim and Protopin. The up-regulated metabolites were (3beta,9xi)-3-(beta-D-Glucopyranosyloxy)-14-hydroxycard-20(22)-enolide and Shikimic Acid in YE988 *vs.* Longkuiza 4, and the down-regulated metabolites were Astragaloside A and 15-epi Prostaglandin A1. The up-regulated metabolites were TG 16:0_18:2_18:2 and 1-(2,4-dihydroxyphenyl)-2-(3,5-dimethoxyphenyl)propan-1-one in YE988 *vs.* Oil Supreme 909S, and the down-regulated metabolite was alpha-Hederin (**Figure 5**). The metabolic pathway annotation found that the DAMs were mainly enriched in Global and overview maps, Carbohydrate metabolism, Biosynthesis of other secondary metabolites, and Amino acid metabolism pathways, that is, the DAMs were mainly enriched in carbon and nitrogen metabolism pathways (**Figure 6**).

### 3.4 KEGG enrichment analysis of differentially abundant metabolites

**Figure 7:**
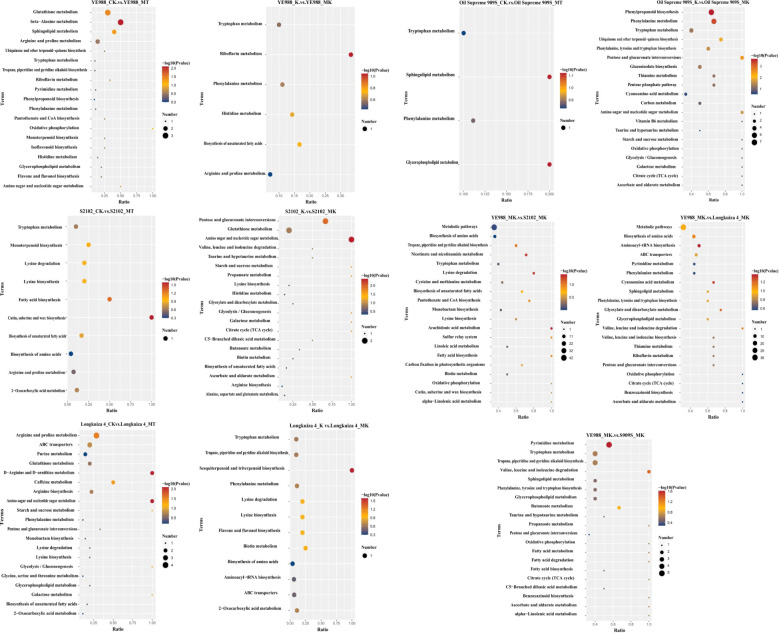
KEGG enrichment analysis of differentially abundant metabolites (DAMs). The abscissa is x/y (the number of DAMs in the metabolic pathway/the number of total metabolites identified in the pathway); The higher the x/y value, the larger the number of DAMs enriched in the pathway. The color of the dots represents the *p* value of the hypergeometric test; The smaller the *p* value, the more statistically significant. The size of the dots represents the number of DAMs enriched in the pathway; The larger the size, the more DAMs enriched in the pathway.

The KEGG enrichment analysis of DAMs showed that in CK *vs.* MT, the DAMs in cultivar YE988 were mainly enriched in Glutathione metabolism, beta−Alanine metabolism, Sphingolipid metabolism, and Arginine and proline metabolism pathways. In K *vs.* MK, the DAMs in cultivar YE988 were mainly enriched in Riboflavin metabolism, Histidine metabolism, and Biosynthesis of unsaturated fatty acids pathways. In CK *vs.* MT, the DAMs in cultivar S2102 were mainly enriched in Cutin, suberine and wax biosynthesis and Fatty acid biosynthesis pathways. In K *vs.* MK, the DAMs in cultivar S2102 were mainly enriched in Amino sugar and nucleotide sugar metabolism, Glutathione metabolism, and Pentose and glucuronate interconversions pathways. In CK *vs.* MT, the DAMs in cultivar Oil Supreme 909S were mainly enriched in Glycerophospholipid metabolism and Sphingolipid metabolism pathways. In K *vs.* MK, the DAMs in cultivar Oil Supreme 909S were mainly enriched in Tryptophan metabolism, Phenylalanine metabolism, and Phenylpropanoid biosynthesis pathways. In CK *vs.* MT, the DAMs in cultivar Longkuiza 4 were mainly enriched in ABC transporters and Arginine and proline metabolism pathways. In K *vs.* MK, the DAMs in cultivar Longkuiza 4 were mainly enriched in Sesquiterpenoid and triterpenoid biosynthesis, Biosynthesis of amino acids, Biotin metabolism, and Flavone and flavonol biosynthesis pathways (**Figure 7**; **Supplementary Figure S3**).

In the MT treatment, the DAMs in YE988 *vs.* S2102 were mainly enriched in Nicotinate and nicotinamide metabolism, Biosynthesis of unsaturated fatty acids, Tropane, piperidine and pyridine alkaloid biosynthesis, and Tryptophan metabolism pathways. The DAMs in YE988 *vs.* Oil Supreme 909S were mainly enriched in ABC transporters, Biosynthesis of amino acids, and Metabolic pathways. The DAMs inYE988 *vs.* Longkuiza 4 were mainly enriched in Biosynthesis of amino acids, Biosynthesis of secondary metabolites, and Metabolic pathways. In the MK treatment, the DAMs in YE988 *vs.* S2102 were mainly enriched in Biosynthesis of amino acids and Metabolic pathways. The DAMs in YE988 *vs.* Oil Supreme 909S were mainly enriched in Tropane, piperidine and pyridine alkaloid biosynthesis, Tryptophan metabolism, and Pyrimidine metabolism pathways. The DAMs in YE988 *vs.* Longkuiza 4 were mainly enriched in Aminoacyl−tRNA biosynthesis, Biosynthesis of amino acids, and Metabolic pathways. Under normal conditions (MT), the stronger the salt resistance of the cultivar, the smaller the number of metabolic pathways enriched by DAMs. Under MK treatment, the stronger the salt resistance of the cultivar, the larger the number of metabolic pathways enriched by DAMs. In the MK treatment, the DAMs in S2102 *vs.* YE988, Longkuiza 4 *vs.* YE988, and Oil Supreme 909S *vs.* YE988 were mainly enriched in pathways such as Tryptophan metabolism, Amino acid biosynthesis, Biosynthesis of secondary metabolites, and Metabolic pathways (**Figure 7**).

### 3.6 Integrated transcriptome and metabolome analysis

**Figure 8:**
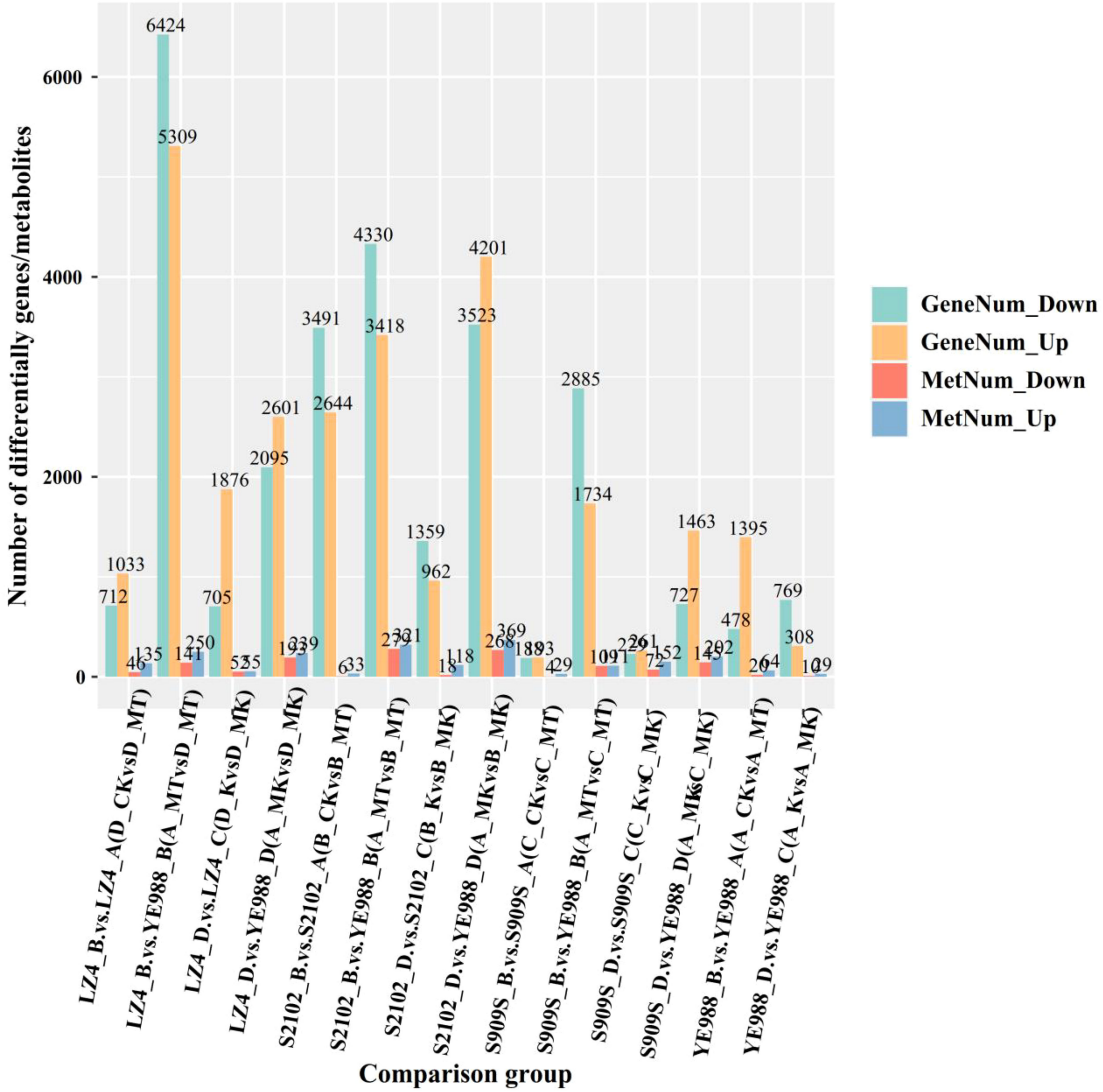
Integrated transcriptome and metabolome analysis. The abscissa represents different *H. annuus* cultivars in different treatments, and the ordinate represents the number of up-regulated and down-regulated genes and metabolites.

The integrated transcriptome and metabolome analysis (**Figure 8**) showed that in the MT treatment, there were 11733 DEGs and 391 DAMs in Longkuiza 4 *vs.* YE988 (6424 down-regulated genes and 5309 up-regulated genes; 141 down-regulated metabolites and 250 up-regulated metabolites), 7748 DEGs and 500 DAMs in S2102 *vs.* YE988 (4330 down-regulated genes and 3418 up-regulated genes; 141 down-regulated metabolites and 221 up-regulated metabolites), 4619 DEGs and 500 DAMs in S909S *vs.* YE988 (2885 down-regulated genes and 1734 up-regulated genes; 108 down-regulated metabolites and 99 up-regulated metabolites). In the MK treatment, there were 4696 DEGs and 432 DAMs in Longkuiza 4 *vs.* YE988 (2095 down-regulated genes and 2601 up-regulated genes; 193 down-regulated metabolites and 239 up-regulated metabolites), 7724 DEGs and 637 DAMs in S2102 *vs.* YE988 (3523 down-regulated genes and 4201 up-regulated genes; 268 down-regulated metabolites and 369 up-regulated metabolites), 2190 DEGs and 342 DAMs in S909S *vs.* YE988 (727 down-regulated genes and 1463 up-regulated genes; 140 down-regulated metabolites and 202 up-regulated metabolites).

**Figure 9:**
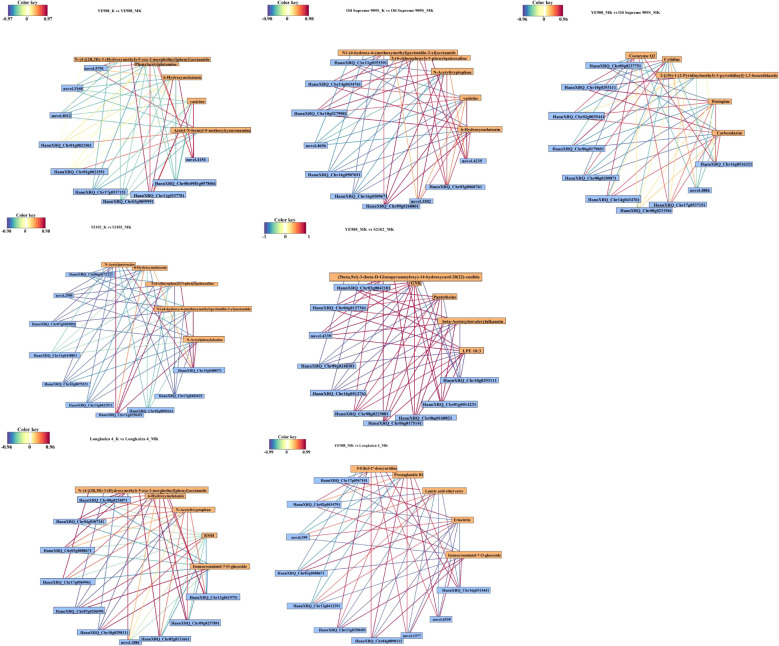
Correlation network of the top ten differentially expressed genes by expression level and the top five differentially abundant metabolites by relative abundance. Yellow boxes represent differentially abundant metabolites, and blue boxes represent differentially expressed genes. Red lines indicate positive correlations, and blue lines indicate negative correlations. The greater the correlation coefficient, the darker the color.

**Figure 10:**
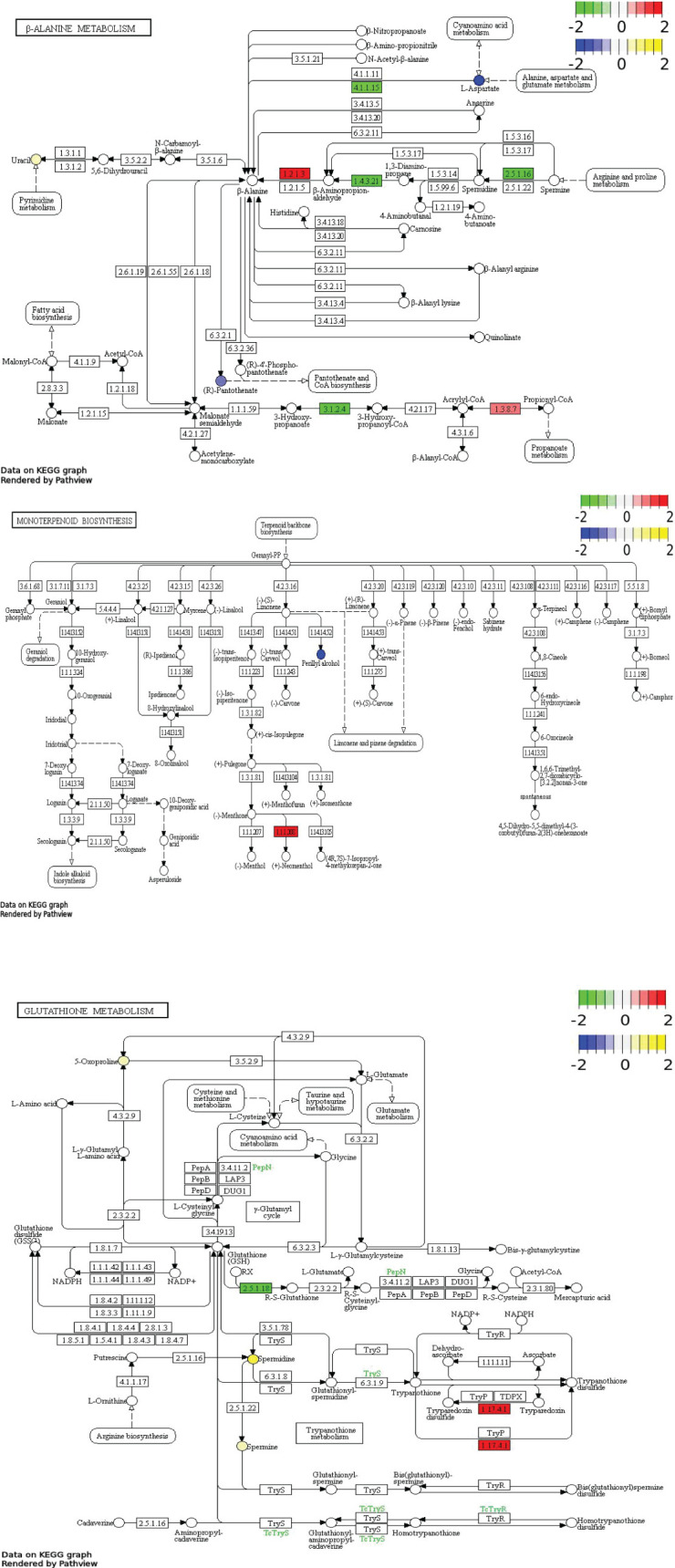
Pathways (β-alanine metabolic pathway; monoterpenoid biosynthesis pathway; glutathione metabolic pathway) enriched by differentially expressed genes (DEGs)/differentially abundant metabolites (DAMs). Blue circles represent down-regulated DAMs, and yellow circles represent up-regulated DAMs. Green boxes represent down-regulated DEGs, and red boxes represent up-regulated DEGs.

Correlation network (**Figure 9**) comprehensively demonstrated the complex interactions between DAMs and DEGs, highlighting the key pathways and regulatory mechanisms involved in melatonin-mediated salt resistance in *H. annuus*. The DAMs and DEGs in MK *vs.* K were mainly enriched in β-alanine metabolism, Monoterpene biosynthesis, and Glutathione metabolism pathways. The KEGG pathway enrichment analysis showed key metabolites and reactions. For example, the glutathione metabolic pathway diagram presents the various steps of glutathione metabolism, related enzymes, intermediate metabolites, and reaction processes. Glutathione could be oxidized to oxidized glutathione (GSSG), under the catalyzing by glutathione peroxidase (GPX). However, GSSG could be reduced to GSH under the action of glutathione reductase (GR) and NADPH. The diagram also shows the correlation between glutathione metabolism and other metabolic pathways, as well as the correlation with amino acid metabolism (such as arginine biosynthesis and tryptophan metabolism). The intermediate products and related enzymes of glutathione metabolism may be involved in other metabolic processes, reflecting the complexity and integrity of the cellular metabolic network (**Figure 10**). The DAMs Spermine, Uracil, Spermidine and the DEGs *HannXRQ_Chr07g0195521*, *HannXRQ_Chr06g0168441*, and *HannXRQ_Chr03g0093321* were mainly involved in β-alanine metabolism. The DAMs Eucalyptol and Geraniol as well as the DEGs *HannXRQ_Chr07g0190521*, *novel.5390*, and *HannXRQ_Chr16g0530401* were mainly involved in Monoterpenoid biosynthesis. The DAMs pyroglutamic acid, spermine, and spermidine were mainly involved in Glutathione metabolism (**Figure 10**).

## 4 Discussion

Shen et al. (2021) found that exogenous melatonin alleviated the salt stress in cotton by regulating the expression of stress-response genes and ion channel genes. Liu et al. (2022) identified 699 DEGs by transcriptome analysis, including 506 up-regulated genes and 193 down-regulated genes, and clarified the mechanism of exogenous melatonin alleviating the salt stress in cucumber. This experiment identified DEGs in *H. annuus* under different treatments. It was found that in MK *vs.* K, there were more DEGs in the MK treatment than in the K treatment. and there were great differences in the number of DEGs between different cultivars within each treatment. In the MK treatment, YE988 *vs.* S2102 had more DEGs than other cultivar comparison groups. This may be due to the fact that these two cultivars have relatively lower salt resistance than other cultivars, thus foliar spraying with melatonin has a more significant impact on their gene expression.

The further GO analysis of the DEGs found that some DEGs directly or indirectly participate in carbon and nitrogen metabolism, β-glucan catabolism, photosynthesis, and other processes. Ren et al. (2022) found that the application of exogenous melatonin up-regulated the expression of genes related to chlorophyll metabolism and photosynthesis in upland cotton seedlings under salt stress, compared with the control. Wei et al. (2019) showed that melatonin treatment could promote the accumulation of glucose, starch, and amino acid metabolites in apple leaves under light stress by regulating the expression of related genes. The results of this study showed that foliar spraying with melatonin promoted the metabolism of linolenic acid in salt-stressed *H. annuus*, and the DEGs in MK *vs.* K were mainly enriched in the linolenic acid metabolism, pentose and glucuronic acid interconversions, and methionine metabolism pathways. Linoleic acid, closely related to photosynthesis, is one of the main fatty acids contained in plant membrane lipids (Liu et al., 2020). Therefore, foliar spraying with melatonin could improve the photosynthesis of salt-stressed *H. annuus* by up-regulating the expression of genes in the linolenic acid metabolism pathway. Chen et al. (2022) also found that salt-resistant *Avena sativa* L. cultivars resisted salt stress mainly by enhancing their carbohydrate and energy metabolism through the pentose-glucuronic acid interconversion pathway. El-Bauome et al. (2022) found that methionine played an important role in the biosynthesis of plant hormone ethylene and cellular metabolism. Therefore, melatonin may enhance the salt resistance of *H. annuus* by regulating the pentose, glucuronic acid, and methionine metabolic pathways.

It was worth noting that in the metabolome analysis, DAMs were mainly enriched in the biosynthesis of amino acids, secondary metabolites, and metabolic pathways of salt-stressed *H. annuus* under melatonin treatment. This was confirmed by the transcriptome analysis results, i.e., the expression of genes related to the synthesis of amino acids such as methionine and the linolenic acid metabolism in salt-stressed *H. annuus* were up-regulated after melatonin treatment. This reveals the key role of amino acid and secondary metabolite synthesis under melatonin treatment. Primary metabolites are metabolites necessary for plant growth, development, and reproduction. In this study, amino acids, as primary metabolites, were significantly affected by melatonin treatment under salt stress. The metabolome analysis found that DAMs were mainly enriched in amino acid metabolism pathways, such as methionine metabolism. Methionine is involved in the biosynthesis of the plant hormone ethylene and various metabolic processes in the cells, playing an important role in plants’ response to salt stress (El-Bauome et al., 2022). Secondary metabolites are unnecessary for plant growth, but play an important role in plants coping with adversity (Al-Khayri et al., 2023). The study found that melatonin treatment regulated the monoterpene biosynthesis pathway, promoting the synthesis of monoterpenes such as eudesmol. Monoterpenes participate in plant defense by regulating membrane stability and alleviating ion toxicity, enhancing plants’ salt resistance (Rai et al., 2023). Primary metabolites are directly involved in osmotic regulation, antioxidation, signaling, and present in all cells. Secondary metabolites are often concentrated in specific organs or tissues and are often regulated by specific genes. They rely on primary metabolites as precursors for synthesis and participating in regulation (Salam et al., 2023).

The results of this study are consistent with previous studies on melatonin-mediated stress resistance (Qian et al., 2023; Sun et al., 2025), and also provide novel insights. Previous research (Zi et al., 2024) has shown that melatonin treatment can enhance stress resistance by regulating metabolic pathways in plants. Based on the integrated transcriptome and metabolome analysis results, the study also revealed three core pathways regulated by melatonin: β-alanine metabolism, Monoterpenoid biosynthesis, and Glutathione metabolism, illustrating a multiple-layer salt resistance system. Firstly, β-alanine plays an important role in the secondary metabolism of plants (i.e., lignin biosynthesis), phospholipid synthesis, fatty acid synthesis and degradation, and tricarboxylic acid cycle (Anutthaman et al., 2019). β-alanine protects plants from abiotic and biotic stresses, and there is evidence that it is involved in lignin biosynthesis and ethylene production in some species (Parthasarathy et al., 2019). In some plants, β-alanine is also converted to β-alanine betaine, an important compound that plays an important role in plants’ resistance to high salinity (Ren and Chen, 2023). The results of this study showed that melatonin could enhance the metabolism of spermine, uracil, and spermine in β-alanine metabolism pathway, as well as the expression of *HannXRQ_Chr07g0195521*, *HannXRQ_Chr06g0168441*, and *HannXRQ_Chr03g0093321*, improving the salt resistance of *H. annuus.* This is confirmed by the results of our another study, i.e., melatonin could increase the free proline content and osmolyte accumulation in salt-stressed *H. annuus* leaves compared with the control (**Supplementary Figure S4**). This is a specific metabolite accumulation pattern that is closely related to stress alleviation. β-alanine is usually associated with secondary metabolism (such as lignin synthesis) in plants, but its function under salt stress has not been clearly associated with melatonin regulation. This finding provides new metabolic targets for studying melatonin-mediated salt resistance.

Secondly, monoterpenoids play a crucial role in plants’ resistance to biotic and abiotic stresses such as drought and salinity (Kleiber et al., 2017; Liu et al., 2023). Studies have shown that terpenoids play an important role in plant photosynthesis by regulating plant growth, development, pollination, and resistance to external biotic and abiotic stresses (Li et al., 2023). Terpenes can serve as essential hormones, protein modifiers, transporters, antioxidants, and photosynthetic pigments for plant growth (Pichersky and Raguso, 2018; Zhou and Pichersky, 2020). Monoterpene volatile organic compounds are highly correlated with plant stress resistance, and monoterpenoids play a crucial role in innate immune signaling in plants (Riedlmeier et al., 2017). The results of this study showed that melatonin could promote the synthesis of metabolites such as eucalyptol and menthol in the Monoterpenoids biosynthesis pathway of salt-stressed *H. annuus*, as well as the expression of *GeraniolHannXRQ_Chr07g0190521*, *novel. 5390*, and *HannXRQ_Chr16g0530401*. Eucalyptol, a type of monoterpenoid, not only participates in plant stress responses, but also alleviate ion toxicity by regulating membrane lipid stability or volatile signaling (Liu et al., 2023). Melatonin treatment enhanced membrane stability and volatile signaling by promoting the biosynthesis of monoterpenes (such as eudesmol and geraniol). This is confirmed by the results of our another study, i.e., melatonin application reduced the content of malondialdehyde in salt-stressed *H. annuus* leaves compared with the control, protecting the cell membrane (**Supplementary Figure S5**). This is a mechanism that has been rarely emphasized in previous melatonin studies. Monoterpenes are usually associated with plants’ insect and disease resistances (Liu et al., 2022). Their role in abiotic stress is a new research direction.

Thirdly, melatonin-induced changes in glutathione metabolism may be the key to enhancing antioxidant defense in *H. annuus* under salt stress. Glutathione is a tripeptide that enhances plant resistance to biotic and abiotic stresses. It can scavenge reactive oxygen species in plant cells under adverse conditions. In addition, glutathione is also involved in plant stress signaling pathways directly (Rai et al., 2023). Previous studies have found that exogenous application of glutathione can increase wheat water use efficiency and antioxidant enzyme activity compared with the control (ur Rehman et al., 2021). Soleh Akram et al. (2017) found that antioxidant enzyme activity and photosynthetic activity were positively correlated with the content of glutathione in plants under salt stress. Ibrahim et al. (2017) found that glutathione application could alleviate the damages of salt stress to cotton seedlings compared with the control. Al-Elwany et al. (2020) found that foliar spraying with exogenous glutathione improved pepper growth and yield under salt stress, and the fruit number per plant and dry fruit weight significantly increased by 235% and 258.1%, respectively compared with those of the control. The results of this study showed that melatonin could increase the relative abundance of metabolites of glutamate, spermine, and spermidine in the glutathione metabolic pathway of *H. annuus* under salt stress. Spermine and spermidine are polyamines closely related to plant resistance to salt stress (Alijani et al., 2024). The melatonin induced changes in glutathione metabolism pathway in this study suggest a defense mechanism that enhances salt resistance in *H. annuus* through redox homeostasis and signal transduction. This is confirmed by the results of our another study, i.e., melatonin application increased the peroxidase activity of *H. annuus* leaves compared with the control, improving the antioxidant capacity (**Supplementary Figure S6**).

Finally, another important insight from this study is the genetic heterogeneity in the responses of different *H. annuus* cultivars to melatonin, which provides a practical direction for precision breeding of salt-resistant cultivars. According to the above molecular mechanisms, β-alanine metabolism, monoterpene biosynthesis, and glutathione metabolism pathways work together to mediate the regulation of melatonin on the salt resistance of *H. annuus*, enabling *H. annuus* plants to maintain their growth and development under salt stress environments. By combining transcriptome and metabolome data, it is suggested that *HannXRQ_Chr07g0195521* and *HannXRQ_Chr03g0093321* can be considered candidate genes for the breeding of salt-resistant *H. annuus* cultivars. This discovery provides support for molecular marker-assisted breeding, i.e., selecting *H. annuus* cultivars with higher expression of these pathways through gene editing or molecular marker. In addition, this study also found significant differences in the response to melatonin application between different *H. annuus* cultivars. Different germplasms exhibited significantly different gene expression and metabolite changes after melatonin treatment. For example, in MK *vs.* K, the lowly salt-resistant cultivars YE988 and S2102 had more DEGs and DAMs than other germplasms after melatonin treatment, while the highly salt-resistant Oil Supreme 909S exhibited stronger metabolic adaptability. Besides, there were also significant differences in the enriched metabolic pathways among different germplasms. After melatonin treatment, the tryptophan metabolism and styrene-like biosynthesis pathways were significantly enriched in Oil Supreme 909S, while Riboflavin metabolism, Histidine metabolism, and Biosynthesis of unsaturated fatty acids pathways were significantly enriched in YE988. These results indicate that the responses to melatonin application may vary significantly depending on genetic background. Therefore, in future agricultural managements, melatonin should be applied according to the characteristics of the cultivar.In addition to the above findings, there are also some novel insights, i.e., the hanges of some key metabolites are related to salt resistance. N-Acetyl-L-carnosine is a kind of carnosine that have antioxidant and anti-inflammatory functions, but its function in plant salt resistance has not yet been clarified (Sultana et al., 2022). In the study, the abundance of N-Acetyl-L-carnosine was significantly down-regulated under salt stress, while melatonin treatment partially restored its level. N-Acetyl-L-carnosine may be involved in salt resistance by regulating intracellular pH or metal ion homeostasis. Coumarins such as fraxinellone have antibacterial and antioxidant functions, but their role in plant salt resistance has been rarely reported (Guilherme, 2013). In the study, the abundance of fraxinellone was significantly down-regulated under salt stress, while melatonin treatment inhibited its downward trend. Therefore, fraxinellone may enhance salt resistance by regulating secondary metabolite balance (Bailly and Vergoten, 2020).

Although this study found that melatonin treatment enhanced *H. annuus* salt resistance by regulating pathways such as β-alanine metabolism, monoterpene biosynthesis, and glutathione metabolism, genes in these metabolic pathways have not been clearly associated with known salt tolerance regulatory networks. It is known that plant salt tolerance regulatory networks involve multiple aspects, including related genes and signaling pathways of ion balance regulation, osmotic regulation, and antioxidant defense. We will further explore the significantly up-regulated or down-regulated transcription factors in response to melatonin under salt stress in the future. Combined with previous study (Sun et al., 2021), it is speculated that these pathways may be regulated by transcription factors such as *MYB*. These transcription factors have been widely reported to participate in salt resistance regulatory networks. In the future, the functions of specific transcription factors will be verified, to further clarify the melatonin-mediated salt resistance mechanism.

Although this study showed that foliar spraying with melatonin could alleviate the salt stress in *H. annuus*, the long-term effect of melatonin treatment still needs to be further explored in the future. In addition, to further confirm the role of melatonin in salt resistance, and promote its application in crop improvement, follow-up experiments can be carried out in terms of gene functional verification, metabolic regulation mechanism, field trial evaluation, etc. For example, gene editing techniques such as CRISPR/Cas9 can be used to knock out or overexpress key genes found in the study (such as *HannXRQ_Chr07g0195521* and *HannXRQ_Chr03g0093321*), to create transgenic plants. Then, under salt stress conditions, the transgenic plants can be compared with wild-type plants, to observe their growth status, physiological indicators, and salt resistance. If the growth of the plants is significantly inhibited after knocking out the key genes, overexpression of these genes may enhance salt resistance. This can strongly confirm the key role of these genes in the melatonin-mediated salt resistance. Quantitative targeted-metabolite profiling can also be carried out to accurately quantify key metabolites in the metabolic pathways (such as β-alanine metabolism, monoterpene biosynthesis, and glutathione metabolism pathways) significantly affected by melatonin treatment, using high-precision detection techniques such as liquid chromatography-tandem mass spectrometry. The dynamic changes of these metabolites under different salt stress levels and melatonin concentrations can be monitored, and the metabolite change curves can be drawn, to deeply explore the interconversion between metabolites and their quantitative relationship with salt resistance. This will help to further understand the mechanisms of melatonin regulating metabolic pathways to enhance salt resistance. Due to the lack of proteomic data, it is impossible to verify the consistency of gene expression and protein abundance. The effects of melatonin on the salt resistance-related proteome changes in *H. annuus* will be further explored in the future.

## 5 Conclusion

Foliar spraying with melatonin significantly changed the expression of some genes and the abundance of metabolites in the carbon and nitrogen metabolism pathways in *H. annuus* leaves under salt stress. The differentially abundant metabolites induced by melatonin were mainly concentrated in the amino acid biosynthesis and metabolism pathways. The integrated transcriptome and metabolome analysis showed that foliar spraying with melatonin significantly up-regulated the β-alanine metabolism, monoterpene biosynthesis, and glutathione metabolism pathways and the expression of *HannXRQ_Chr07g0195521* and *HannXRQ_Chr03g0093321* in *H. annuus* leaves under salt stress. This further led to an increase in the content of metabolites such as spermine and spermidine, improving the salt resistance of *H. annuus*. This study reveals the genes and metabolic pathways related to salt resistance in *H. annuus*, providing a scientific basis for the precise selection and breeding of *H. annuus* cultivars with stronger salt resistance using modern biotechnology in the future.

## Data Availability

The raw data supporting the conclusions of this article will be made available by the authors, without undue reservation.
