# Supplementary material for: Transcriptome and metabolome analysis of the responses of salt resistance of different Helianthus annuus germplasms to melatonin
Source: Front Plant Sci. 2025 Apr 16;16:1558877. doi: 10.3389/fpls.2025.1558877 (PMC12040833; doi:10.3389/fpls.2025.1558877)
Supplement: Supplementary file 1 [file DataSheet1.docx]

Table S1 Analysis of variance of physiological parameters related to salt resistance of different *Helianthus annuus* varieties during the seedling stage.

| Physiological parameter | Coefficient of variation | Mean square error | *F* value |
| --- | --- | --- | --- |
| Superoxide dismutase activity | 51.09% | 173021.64 | 2194.05** |
| Malondialdehyde content | 57.26% | 7.29 | 82.12** |
| Catalase activity | 261.32% | 2502108.27 | 146.68** |
| Peroxidase activity | 167.78% | 88138924.30 | 20.29** |
| Free proline content | 137.64% | 57651152.09 | 36.41** |

Note: **, *p* < 0.01.

Table S2 Effects of different concentrations of melatonin on the germination rate of *Helianthus annuus* seeds under salt stress.

| Salt solution (mol·L^-1^) | Melatonin solution (μmol·L^-1^) | Germination rate (%) |
| --- | --- | --- |
| 0.0796 | 0 | 28 |
|  | 1 | 32 |
|  | 10 | 40 |
|  | 50 | 36 |
|  | 100 | 32 |
|  | 150 | 36 |
|  | 200 | 32 |

Table S3 Structural variations and annotation.

| HROM | POS | ID | REF | ALT | QUAL | DP | AD | GT | GeneID | GeneName | FeatureID | Biotype | IMPACT |
| --- | --- | --- | --- | --- | --- | --- | --- | --- | --- | --- | --- | --- | --- |
| 1 | 623876 | . | T | G | 208.6 | 14 | 7,7 | 0/1 | HannXRQ_Chr01g0000081 | HannXRQ_Chr01g0000081 | HannXRQ_Chr01g0000081-1 | lincRNA | MODIFIER |
| 1 | 624063 | . | A | C | 562.6 | 37 | 20,17 | 0/1 | HannXRQ_Chr01g0000081 | HannXRQ_Chr01g0000081 | HannXRQ_Chr01g0000081-1 | lincRNA | MODIFIER |
| 1 | 624257 | . | G | A | 686.6 | 30 | 12,18 | 0/1 | HannXRQ_Chr01g0000081 | HannXRQ_Chr01g0000081 | HannXRQ_Chr01g0000081-1 | lincRNA | LOW |
| 1 | 626193 | . | A | G | 323.61 | 10 | 1,8 | 0/1 | HannXRQ_Chr01g0000081 | HannXRQ_Chr01g0000081 | HannXRQ_Chr01g0000081-1 | lincRNA | MODIFIER |
| 1 | 1031828 | . | T | C | 93.6 | 11 | 8,3 | 0/1 | HannXRQ_Chr01g0000101 | HannXRQ_Chr01g0000101 | OTG35745 | protein_coding | MODIFIER |

Notes: CHROM, Number of chromosome where the structural variation is located; POS, Coordinate of chromosome where the structural variation is located; ID, Number of structural variation, which corresponds to the ID in the dbSNP database. If there is no correspondence, the default is to use ‘.’; REF, The base of the structural variation in the reference genome; ALT, The actual base of the structural variation in the sample; QUAL: The quality of the structural variation. The values are in Phred format, and the higher the value, the more reliable the genotype where the structural variation is located; DP, Sequencing depth of the structural variation in the sample; A,D, The number of reads supporting REF and ALT separately; GT, Genotype of the structural variation; GeneID, Number of the gene where the structural variation is located; GeneName, Name of the gene where the structural variation is located; FeatureID, Number of the transcript where the structural variation is located; Biotype, Type of transcript where the structural variation is located; IMPACT, Degree of impact caused by the structural variation.


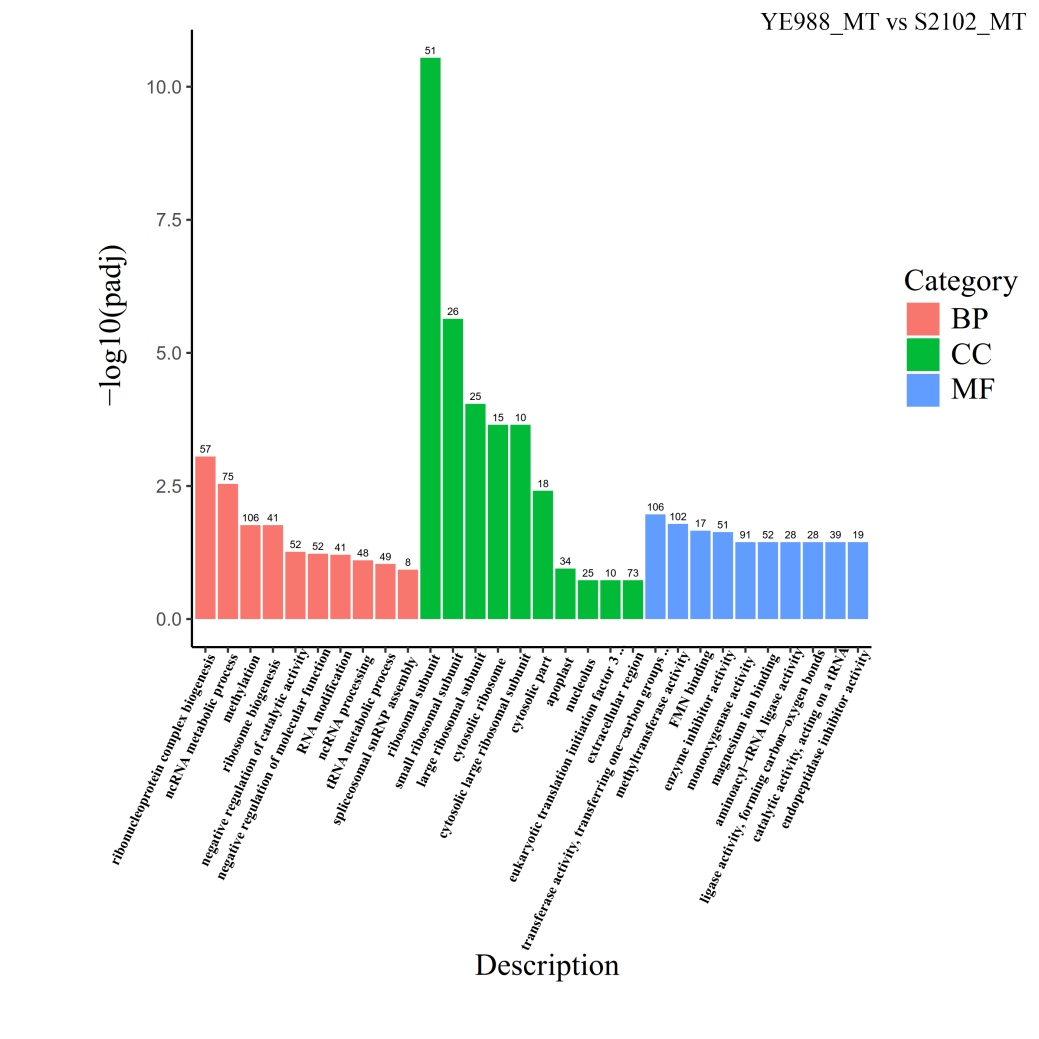

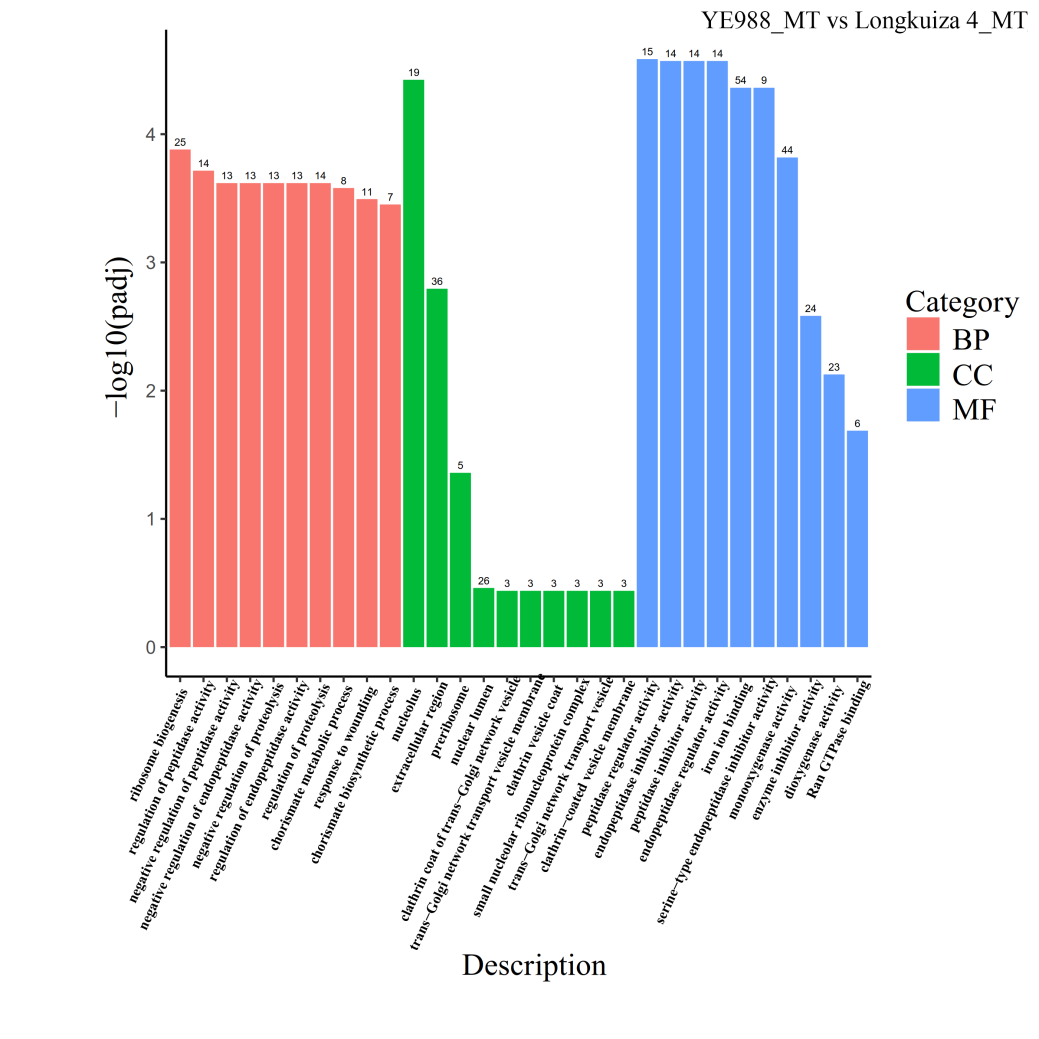

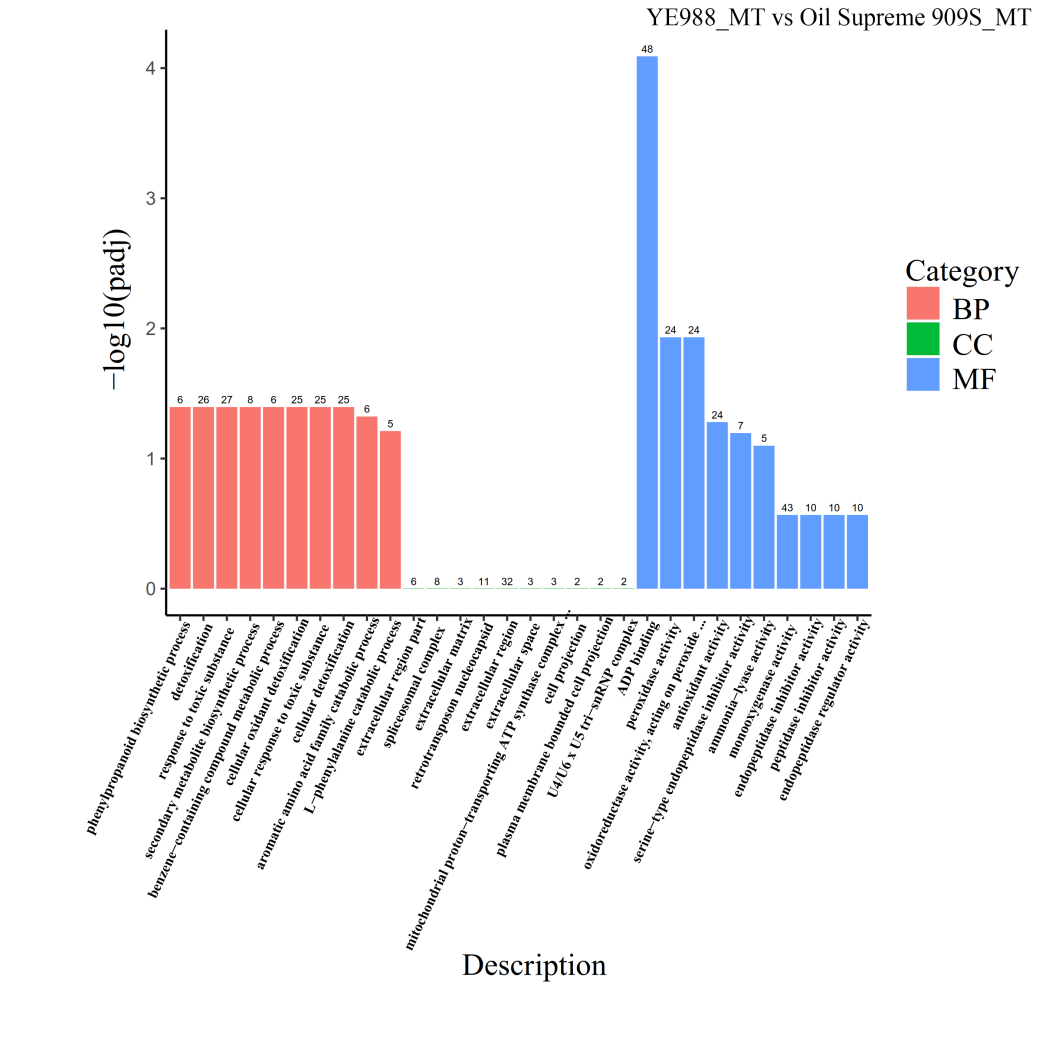


Fig. S1 Gene ontology (GO) analysis of differentially expressed genes (DEGs). The abscissa represents GO terms, and the ordinate represents the significance level of GO term enrichment, with higher values indicating greater significance. The values on the column represented the number of enriched DEGs.


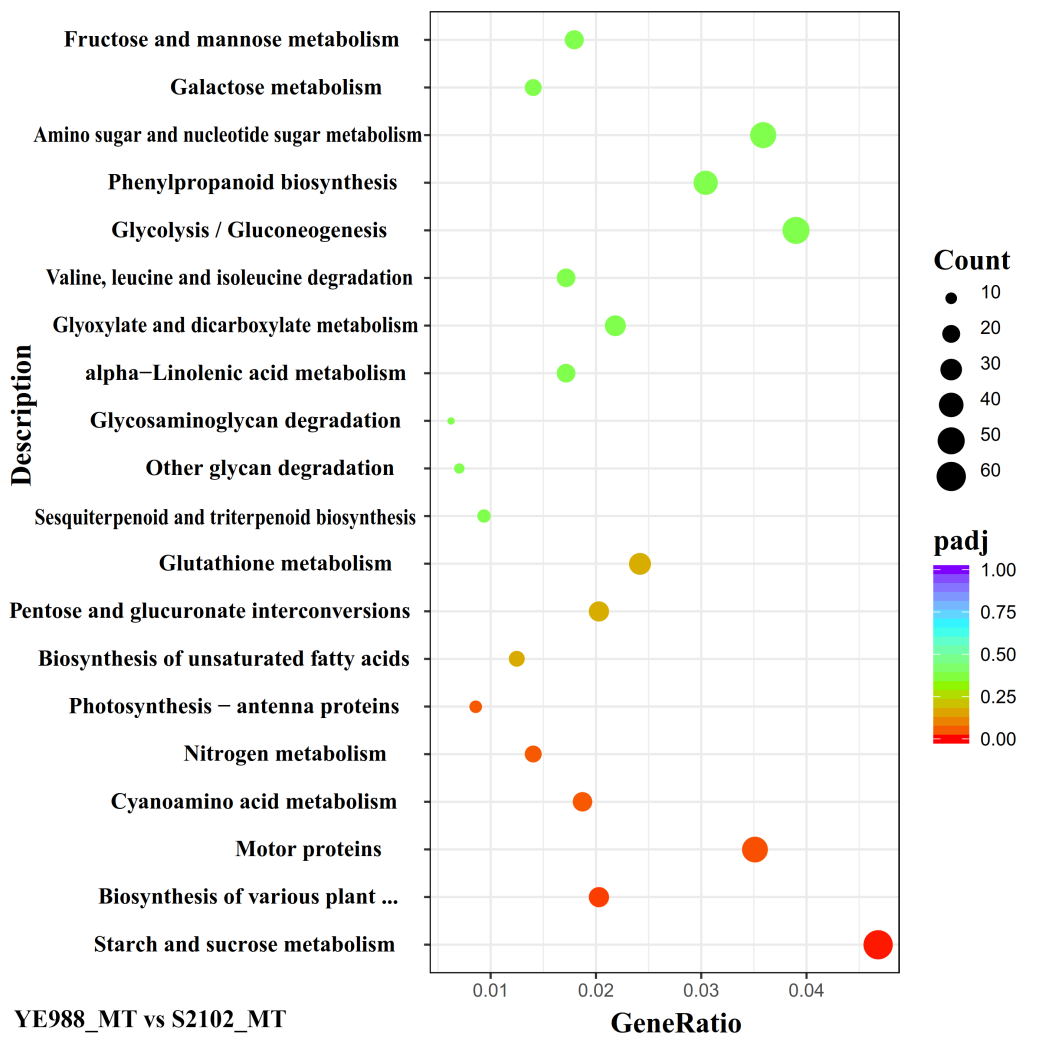


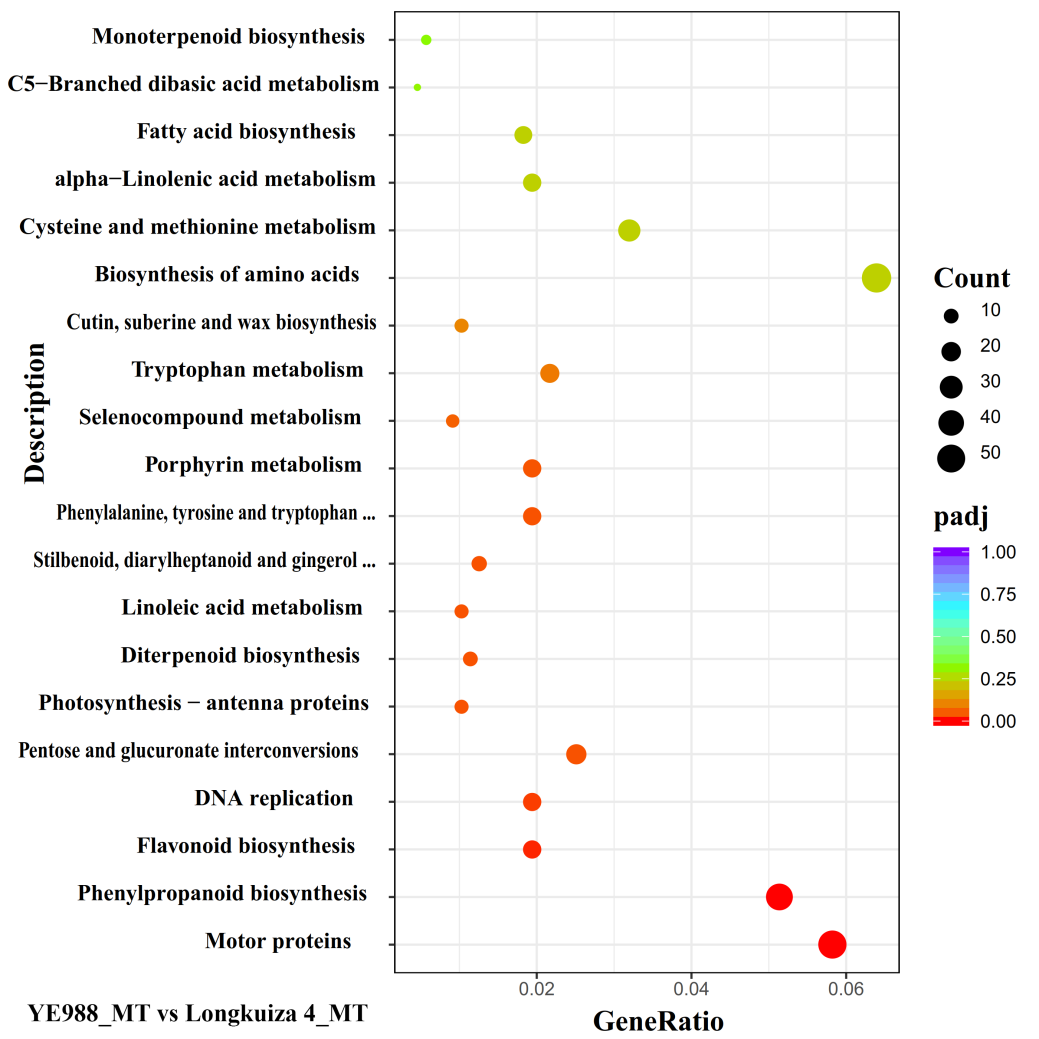


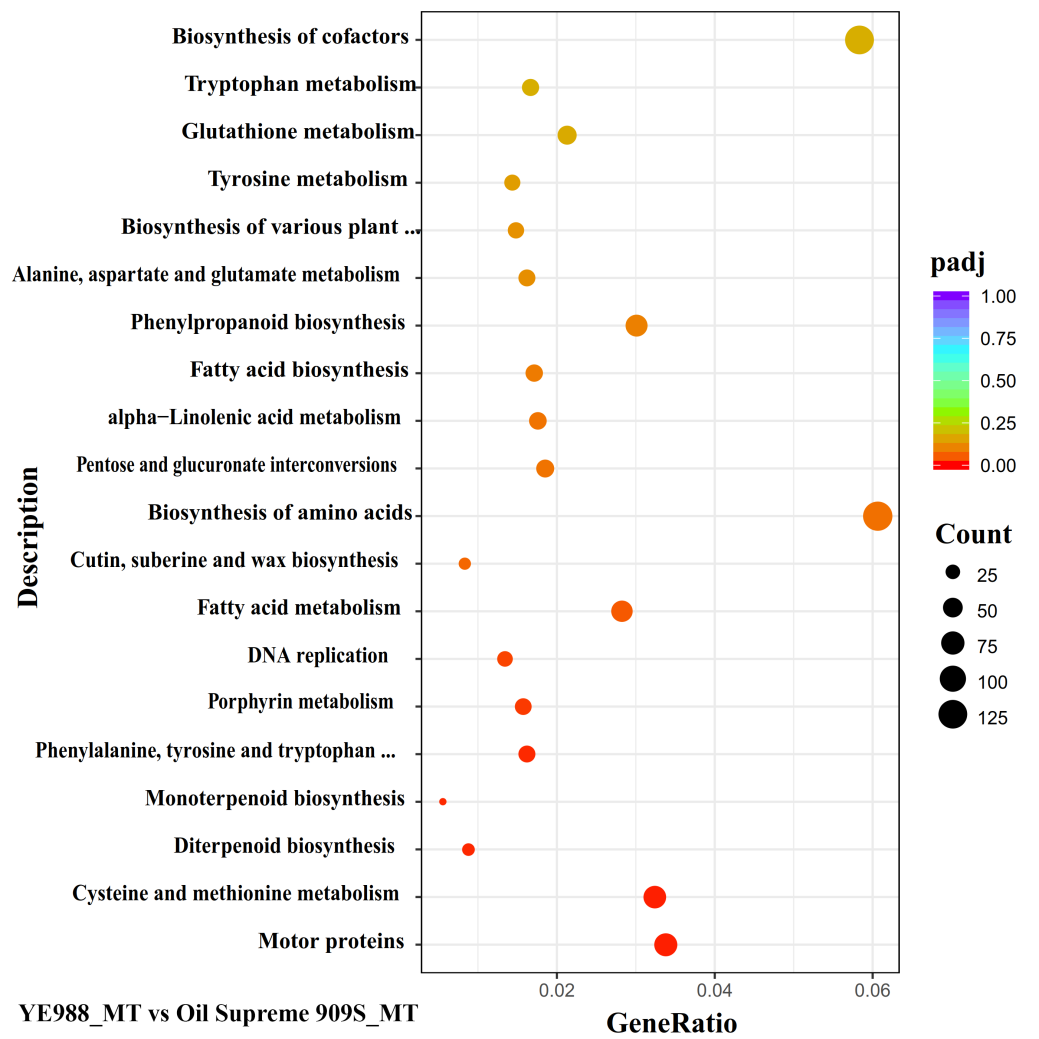


Fig. S2 Kyoto Encyclopedia of Genes and Genomes (KEGG) enrichment analysis of differentially expressed genes (DEGs). The abscissa represents the ratio of the number of DEGs annotated on GO (Gene ontology) terms to the total number of DEGs, and the ordinate represents GO terms. The size of the dots represents the number of genes annotated on GO terms, and the colors from red to purple represent the significance of enrichment from high to low.


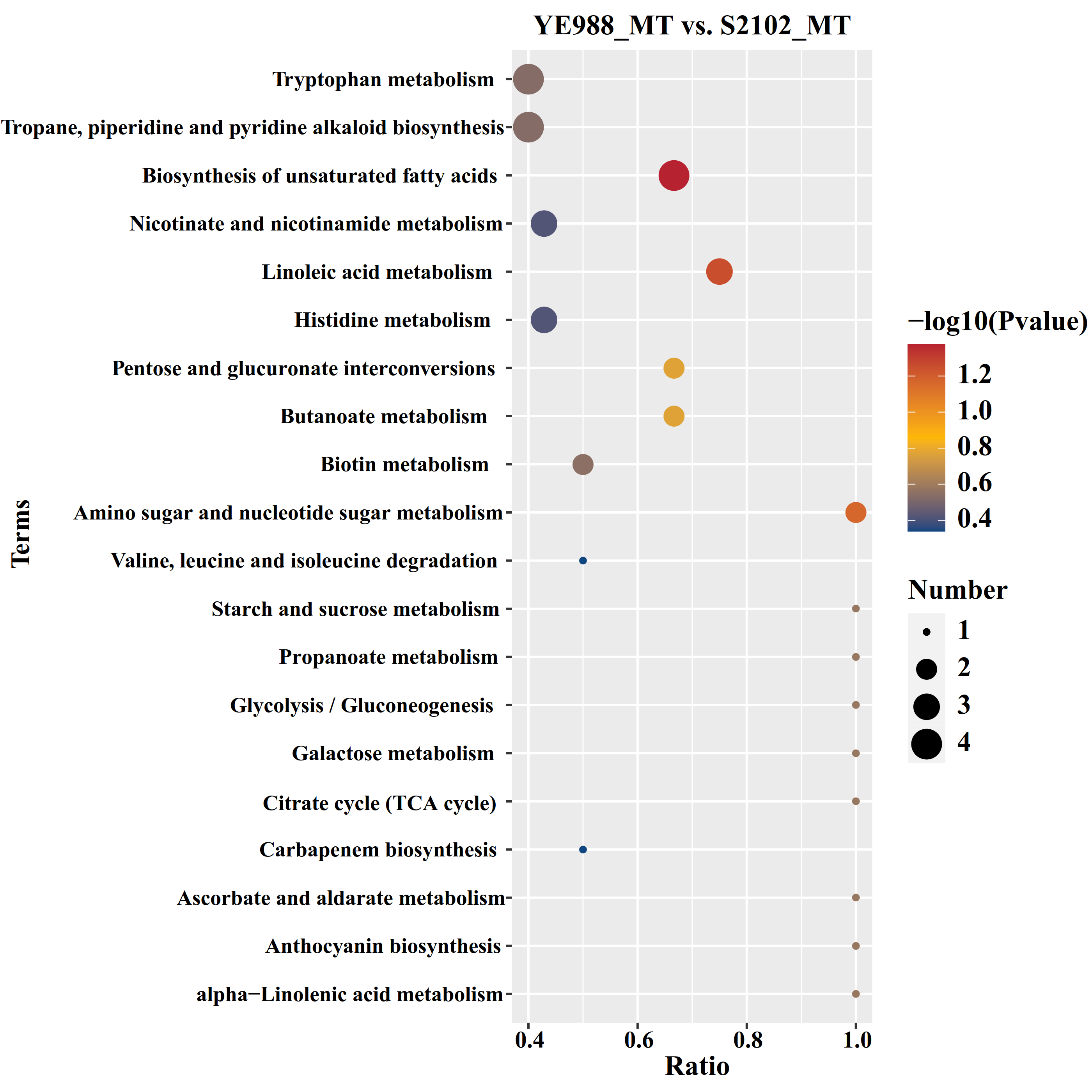

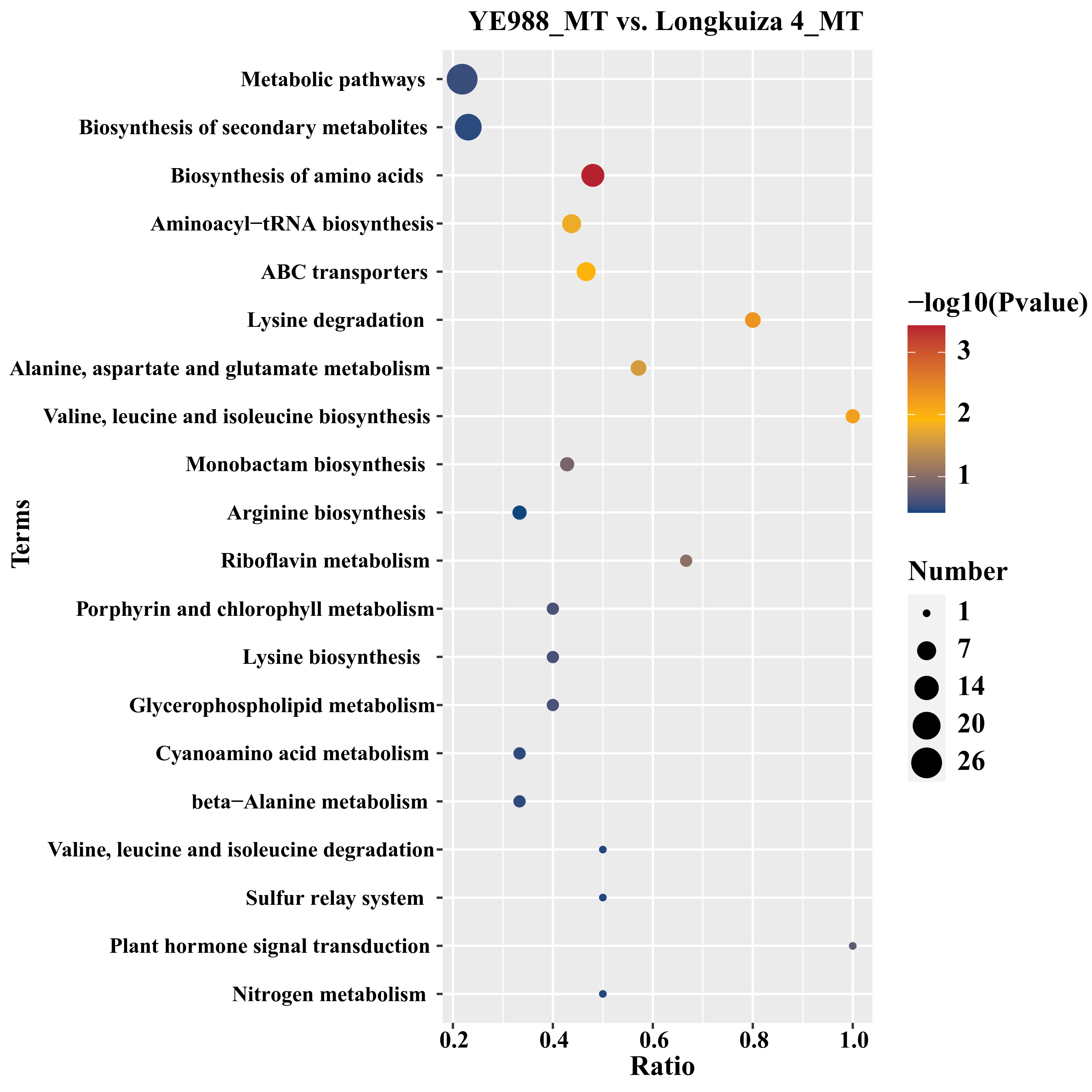


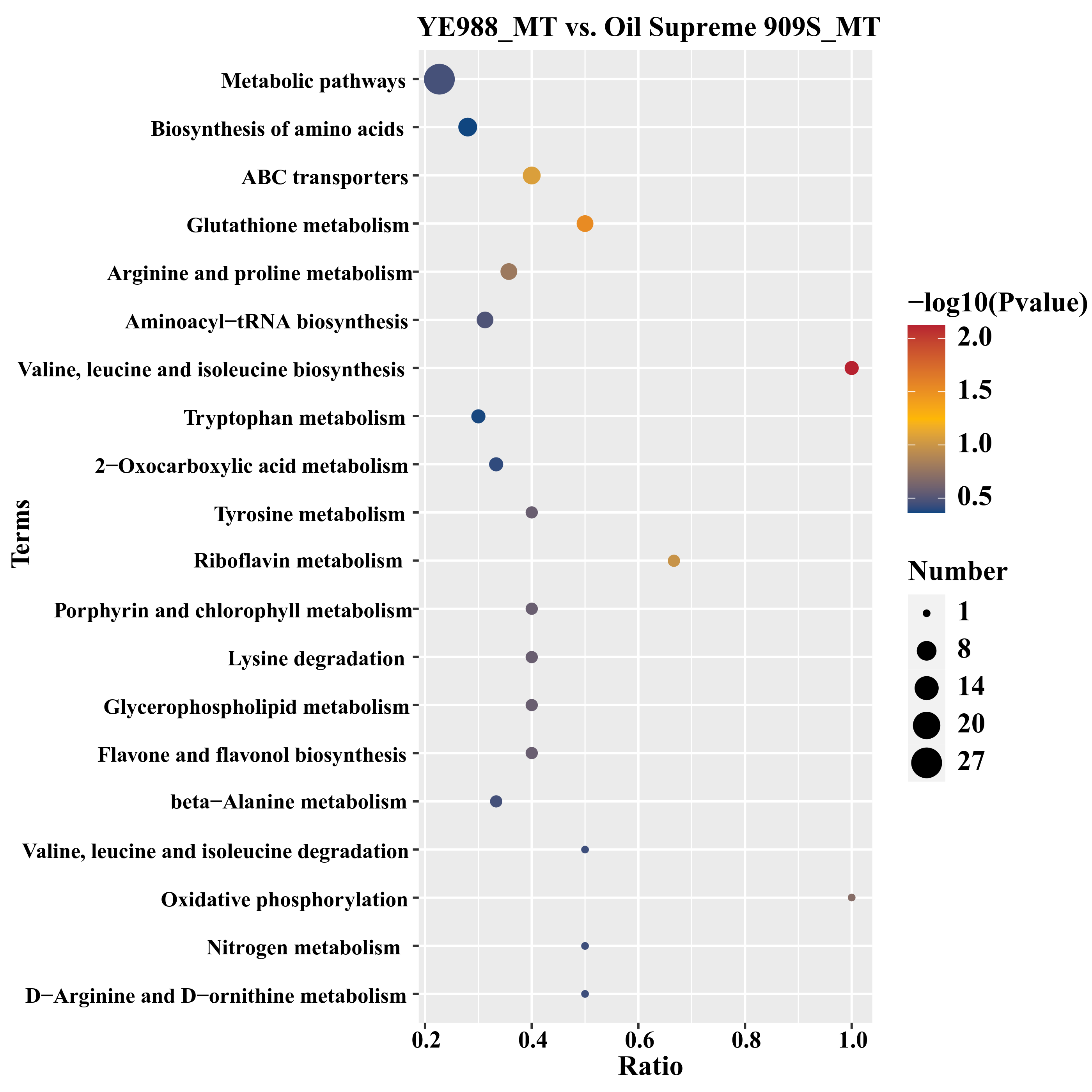


Fig. S3 KEGG enrichment analysis of differentially abundant metabolites (DAMs). The abscissa is x/y (the number of DAMs in the metabolic pathway/the number of total metabolites identified in the pathway); The higher the x/y value, the larger the number of DAMs enriched in the pathway. The color of the dots represents the *p* value of the hypergeometric test; The smaller the *p* value, the greater the reliability and the more statistically significant. The size of the dots represents the number of DAMs in the pathway; The larger the size, the more DAMs in the pathway.


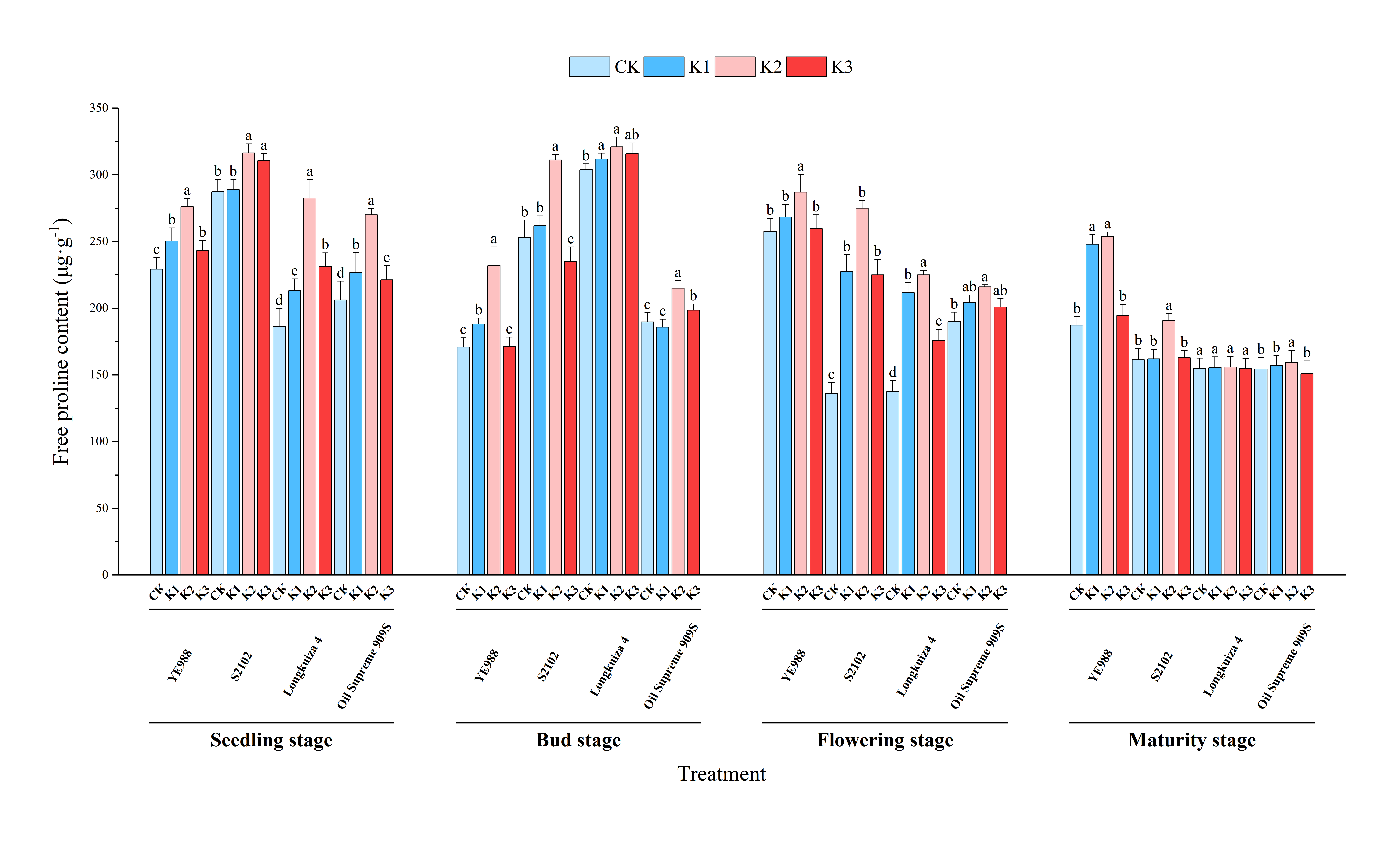


Fig. S4 Effect of exogenous melatonin treatment on free proline content of sunflower under saline-alkali stress.CK, K1 K2, and K3 represent melatonin treatments at concentrations of 0, 1, 10, and 100 μmol·L^-1^, respectively. Different lowercase letters indicate significant difference between treatments at *p* < 0.05. The same below.


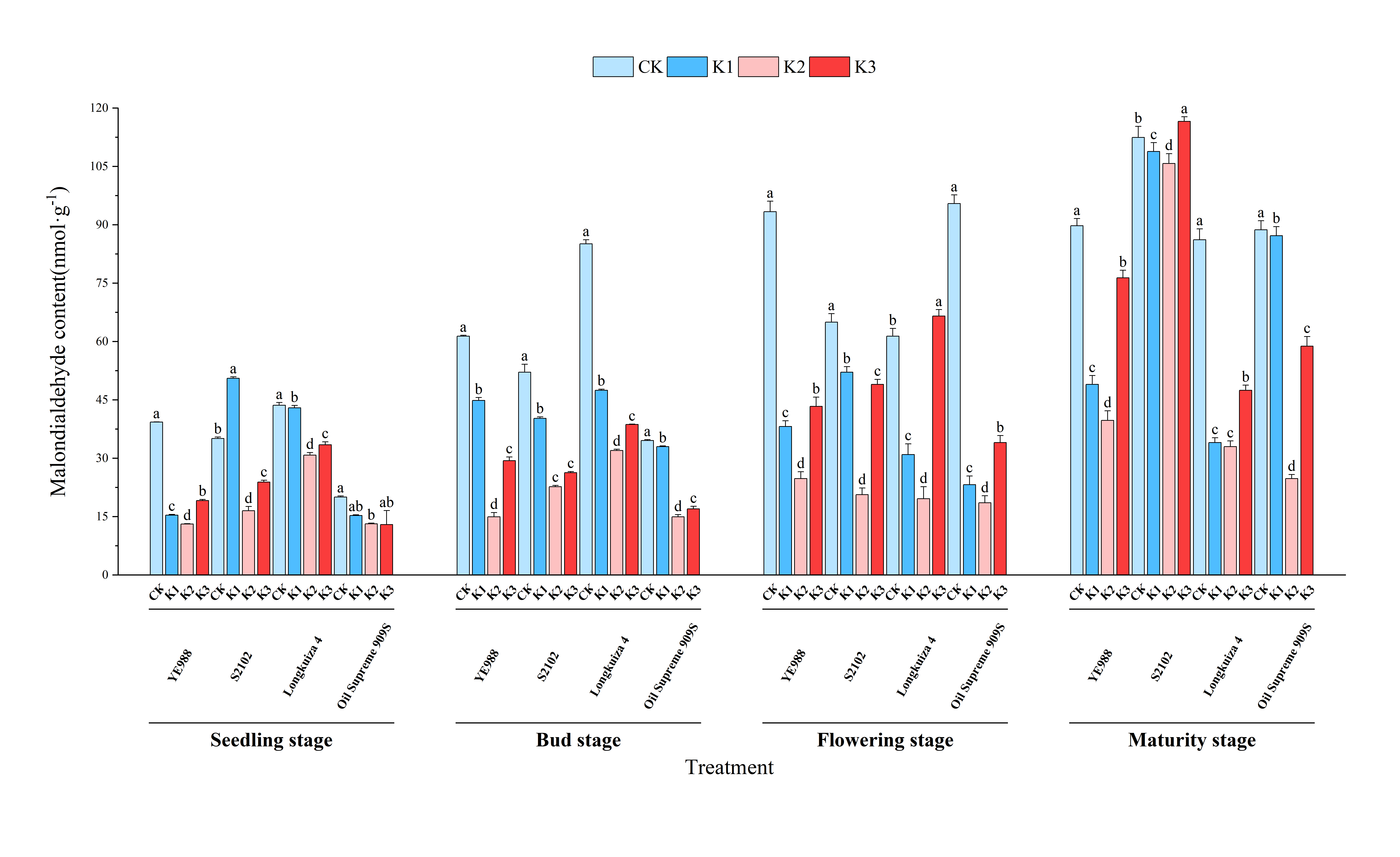


Fig. S5 Effects of exogenous melatonin treatment on malondialdehyde content in sunflower under saline-alkali stress.


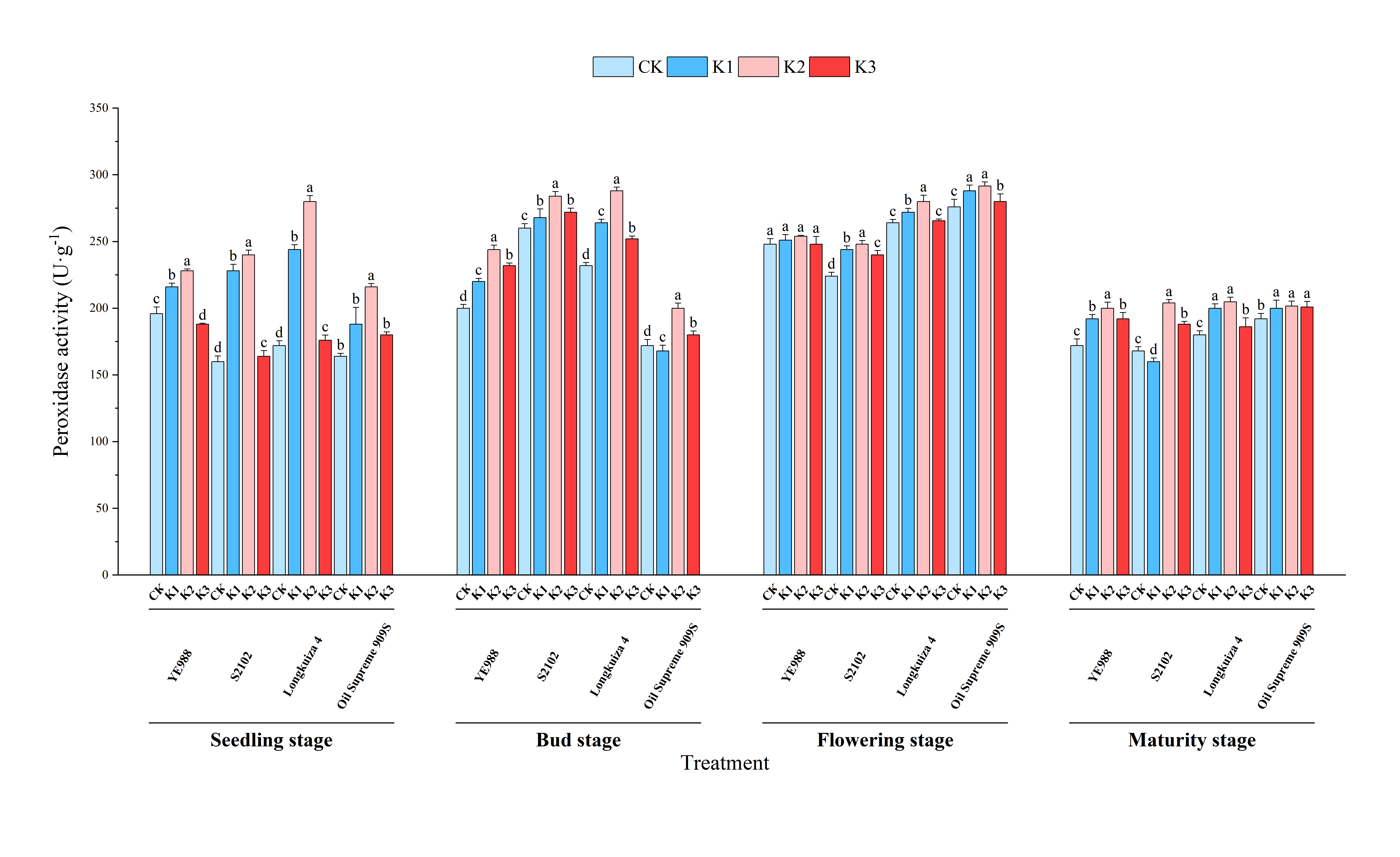


Fig. S6 Effects of exogenous melatonin treatment on peroxidase ( POD ) activity of sunflower under saline-alkali stress.
